# Untargeted metabolomic and transcriptomic analysis in spring and durum wheat reveals potential mechanisms associated with the early stem solidness phenotype and resistance to wheat stem sawfly

**DOI:** 10.3389/fpls.2025.1497732

**Published:** 2025-02-19

**Authors:** Megan S. Hager, Jason P. Cook, Brian Bothner, David K. Weaver

**Affiliations:** ^1^ Wheat Stem Sawfly Laboratory, Department of Land Resources and Environmental Sciences, Montana State University, Bozeman, MT, United States; ^2^ Department of Plant Sciences and Plant Pathology, Montana State University, Bozeman MT, United States; ^3^ Department of Chemistry and Biochemistry, Montana State University, Bozeman MT, United States

**Keywords:** wheat stem sawfly, host plant resistance, metabolomics, transcriptomics, *Triticum aestivum*, *Triticum turgidum* L. var *durum*

## Abstract

Wheat stem sawfly (WSS) causes devastating yield loss in both common bread wheat (*Triticum aestivum* L.) and durum wheat (*Triticum turgidum* L. var *durum*) in the North American Great Plains. The early stem solidness phenotype confers solid stems early in plant development coinciding with the flight period of WSS and provides protection to plants during the critical oviposition period. With this phenotype, pith is lost as the plant develops, which may allow for enhanced biological control of surviving larvae by braconid parasitoids *Bracon cephi* (Gahan) and *Bracon lissogaster* Muesebeck, as well as having additional potential yield benefits from utilizing reabsorbed pith components. Here, we use an untargeted transcriptomics and metabolomics approach to explore the mechanisms related to the early stem solidness phenotype in three cultivars of spring wheat and two cultivars of durum wheat in addition to three near- isogenic pairs of spring wheat and two near- isogenic pairs of durum wheat. We identified effects of growth stage and allele on expression of metabolites and transcripts associated with stem solidness, development of cell walls and programmed cell death. A caffeic acid methylesterase and pectin methylesterase were upregulated in hollow stemmed Reeder and lines with the *3BLa* allele, which likely influences lignin subunit proportions as well as the production of volatile semiochemicals that impact the behavior of adult WSS. *TaVPE3cB*, a gene associated with programmed cell death and thickening of cell walls, also had increased expression in hollow stemmed lines and is likely partially responsible for the hollow stemmed phenotype observed. Growth stage and allele also affected the expression of transcripts and metabolites involved in the phenylpropanoid pathway, carbohydrate and glycoside biosynthesis and lipid biosynthesis, implicating the involvement of these pathways in resistance and plant response to infestation by WSS.

## 1 Introduction

Wheat stem sawfly (WSS), *Cephus cinctus* Norton, is a major pest of both common bread wheat *Triticum aestivum* L., and durum wheat *Triticum turgidum* L. var *durum*, causing up to $350 million in yield loss annually in the Great Plains of North America (Beres et al., 2011; Weaver, 2023). Adult WSS emerge from the stubble of the previous year in late spring or early summer over the period of several weeks (Wallace and McNeal, 1966). Shortly after emerging, WSS females oviposit in the wheat stem and larvae feed inside the stem until they are fully mature. Mature larvae prepare for diapause by cutting a ring around the inner stem wall at the base of the plant, plugging the hole with frass and forming a cocoon inside the space, allowing them to overwinter in the stubble below the soil surface (Beres et al., 2007). Feeding activity of the larvae damages vascular tissues and negatively affects photosynthetic ability, leading to decreased head weight and overall yield loss (Delaney et al., 2010; Macedo et al., 2005). Additionally, stem-cutting by the larvae weakens stems and causes them to lodge when exposed to wind or gravity, making harvesting difficult and leading to further yield loss (Ainslie, 1920; Beres et al., 2007; Morrill et al., 1998; Sherman et al., 2010).

Control of WSS is challenging for several reasons including the protracted flight period of adult WSS, location of the larvae within the stem and unreliable biological control options. Since the larvae are protected inside the stem and adults emerge over a period of weeks, it is difficult to optimally time application of contact insecticide sprays and single applications are often ineffective. There are currently no systemic insecticides labeled for WSS control. In wheat fields, biological control exists in the form of two species of braconid wasps that are known to frequently parasitize WSS larvae. These parasitoids, *Bracon cephi* (Gahan) and *Bracon lissogaster* Muesebeck (Hymenoptera: Braconidae), reduce yield loss by increasing larval mortality (Buteler et al., 2008; Bekkerman and Weaver, 2018). Rates of parasitism from year to year are often inconsistent and can be negatively affected by the use of other WSS control methods including the planting of solid stemmed cultivars, making it an unreliable source of WSS control (Runyon, 2001).

Historically, the use of solid stemmed cultivars has been proven as a partially effective management strategy for WSS. The internodal lacuna of solid stemmed cultivars is filled with parenchyma tissue called pith (Bathini et al., 2023). Solid stems are thought to slow or stop larval growth and maturation by restricting the movement of the larvae within the stem (Holmes and Peterson, 1961, 1962; Bathini et al., 2023; Delaney et al., 2010; Peirce et al., 2022). These cultivars experience lower rates of stem cutting and have the potential to reduce WSS populations over time compared to hollow stemmed cultivars (Bekkerman and Weaver, 2018; Sherman et al., 2015; Peirce et al., 2022). However, solid stems do not provide complete resistance to WSS infestation and stem cutting of 30% or more can still occur in fields planted with solid stemmed cultivars (Talbert et al., 2014). In addition, producers are sometimes reluctant to plant these cultivars as they are perceived to be lower yielding since they are thought to allocate resources to pith production that could otherwise be utilized in grain fill (Beres et al., 2017; Weaver, 2023). These limitations of solid stemmed cultivars make the early stem solidness phenotype of particular interest, since stem solidness is expressed early in plant development but disappears as the plant matures. This provides the plant with necessary protection against infestation by WSS during early development but allows for re-allocation of resources during the critical stage of grain fill. Associated with the allele *Qss.msub-3BLc* in spring wheat, and *Qss.msub-3AL.b* in durum wheat, this development coincides with the life cycle of the WSS, providing plants with protection against infestation only during the most critical stage of growth (Varella et al., 2015, 2019). Despite extensive research on the solid stem phenotype, the genetic basis for the early stem solidness phenotype and the molecular mechanisms involved have not yet been explored prior to this study.

In spring wheat, the stem solidness trait is primarily associated with *Qss.msub-3BL*, a quantitative trait locus (QTL) located on the long arm of chromosome 3B which contributes 76% of the total variation for stem solidness (Cook et al., 2004). Durum wheat shares a common locus on chromosome 3B that is also associated with stem solidness, designated *SSt1* (Houshmand et al., 2007). Despite this shared locus, expression of the stem solidness phenotype differs between durum and spring wheat (Nilsen et al., 2017). In spring wheat, three alleles have been identified at this QTL: the Reeder (PI613586) (Sherman et al., 2010) allele *Qss.msub-3BLa*, which is associated with a hollow stem phenotype (Varella, 2016; Cook et al., 2019); the Choteau (PI633974) allele *Qss.msub-3BLb*, historically called the Rescue allele, which is associated with solid stems throughout development; and the Conan (PI607549) (WestBred, LLC) allele *Qss.msub-3BLc*, which is associated with stem solidness early in plant development. Near isogenic lines with a common genetic background that differed at the 3B QTL were developed by the Spring Wheat Breeding Program at Montana State University to study the effect of this QTL on agronomic traits. The Conan allele *Qss.msub-3BLc* confers stem solidness at early stages in plant development, which disappears as the plant matures (Varella, 2016). This temporal expression of solid stems coincides with the time of year when WSS females are actively laying eggs and larvae are beginning to feed. Resistance due to this allele occurs both through antibiosis, by slowing larval development and causing increased larval mortality as well as through antixenosis, by causing WSS females to make fewer ovipositor insertions and lay fewer eggs (Varella et al., 2015).

In a durum wheat mapping population, a QTL was identified which was associated with early stem solidness and resistance on chromosome 3A, and near isogenic lines were developed from the resistant cultivar Pierce (PI 632366) and the susceptible landrace PI 41353 (PI 41353) (Varella et al., 2019b). Early stem solidness was observed in lines with the Pierce allele (*Qss.msub-3AL.b*), and lines with this allele also experienced a 25% reduction in stem cutting. PI 41353 contributed the *Qss.msub-3AL.a* allele associated with this higher incidence of stem cutting. More stem boring was observed with this allele as well, meaning that larval mortality does not happen as quickly as it does in plants with the Pierce allele (Varella et al., 2019b). In hexaploid spring wheat, a QTL on chromosome 3A was also found to be associated with larval mortality (Varella, 2016).

“Omics” technologies used to study gene expression and environmental responses have broad implications for the future of genome research in many crops, including tetraploid and hexaploid wheat, but these methods have not been used extensively to study Montana cultivars developed for management of WSS. Transcriptomics, or analysis of the gene transcripts in a system, can offer a picture of which genes may be involved in plant response to abiotic and biotic stresses, and has already been used to study several aspects of WSS resistance (Li et al., 2021b; Biyiklioglu et al., 2018; Zhou et al., 2011; Zhao, 2019). A comparison of hollow and solid NILs of spring wheat revealed differential expression of a gene linked to *Qss.msub.3BL* that is involved in lignin biosynthesis (Oiestad et al., 2017). Analysis of the transcriptome of spring wheat cultivars infested with WSS have also identified the involvement of the phenylpropanoid and phosphate pentose pathways in plant defense against larval feeding (Biyiklioglu et al., 2018). So far, studies have not been performed to identify sources of inherent resistance associated with the early stem solidness trait. Metabolite analysis, or metabolomics, can also be used to obtain a more complete picture of the physiological differences between resistant and susceptible plants. These compounds are involved in every aspect of plant function and change rapidly in response to environmental changes, making them a perfect target to obtain a “molecular snapshot” of the state of the plant at a specific point in time. WSS infestation has been found to cause changes in the metabolome of several spring wheat cultivars, particularly in the alkaloid, benzenoid and lipid chemical classes (Lavergne et al., 2020). In oat (*Avena sativa* L.), a species resistant to WSS infestation, infested plants also experienced an increase in lipids and plant defense compounds such as benzoxazinoids compared to control plants (Hager et al., 2024). Differences in the metabolite profiles of solid stemmed and hollow stemmed durum wheat lines have also been observed, including increased levels of osmolytes in hollow stemmed lines and variation in the concentrations of water-soluble carbohydrates in the absence of WSS infestation (Nilsen et al., 2020).

Solid stems do not completely protect against WSS infestation, and braconid parasitoids are not as effective as they are in hollow stemmed cultivars, which makes it difficult to incorporate solid stemmed cultivars as part of a holistic management strategy (Buteler et al., 2015; Rand et al., 2012, 2020). Early stem solidness alleles *Qss.msub-3BLc* in common wheat and *Qss.msub-3AL.b* in durum wheat both lead to decreased infestation and stem cutting, with little effect on other agronomic traits, making these alleles of particular interest for the exploration of related resistance mechanisms and for the development of resistant cultivars (Varella et al., 2015, 2019; Sherman et al., 2015). In this study, transcriptomics and metabolomics will be used to gain a more comprehensive understanding of mechanisms of resistance to WSS and will be used to further explore plant resistance to WSS associated with the early stem solidness phenotype.

## 2 Materials and methods

Spring wheat cultivars Reeder and Conan and near isogenic lines (NILs) developed from these two recurrent parents were used for this study (**Table 1**). Reeder is a hollow stemmed variety, containing the *Qss.msub-3BLa* allele while Conan exhibits early stem solidness associated with the *Qss.msub-3BLc* allele. The near-isogenic lines used in this study were developed by the spring wheat breeding program at Montana State University. Two NIL pairs for the 3B QTL were also used, these consisted of a resistant line with the *Qss.msub-3BLc* allele which confers early stem solidness and a susceptible line with allele *Qss.msub-3BLa* which exhibits a hollow stemmed phenotype. For the 3A QTL, Pierce, a durum wheat with early stem solidness and PI 41353, a hollow stemmed variety were used, as well as a durum wheat NIL pair with a resistant line containing the Pierce allele, *Qss.msub-3AL.b* for stem solidness and a susceptible line with the PI 41353 allele *Qss.msub-3AL.a* (**Table 1**).

**Table 1:** Near isogenic lines used in this study.

Species	Name	Pedigree	Allele
Spring wheat	Reeder	IAS#4/H567.71//Stoa/3/ND674	*3BLa* (hollow)
Spring wheat	Conan	WestBred Rambo/WestBred 906R	*3BLc* (early solid)
Spring wheat	H *3BLc*	Vida X Conan (BC3F2)	*3BLc* (early solid)
Spring wheat	I *3BLb*	Vida X Conan (BC3F2)	*3BLb* (solid)
Spring wheat	J *3BLc*	VidaPsP3 X Conan (BC3F2)	*3BLc* (early solid)
Spring wheat	K *3BLa*	VidaPsP3 X Conan (BC3F2)	*3BLa* (hollow)
Spring wheat	L *3BLa*	WHIT6*6/Choteau	*3BLa* (hollow)
Spring wheat	M *3BLb*	WHIT6*6/Choteau	*3BLb* (solid)
Durum wheat	PI 41353	Landrace (India)	*3ALa* (hollow)
Durum wheat	Pierce	D86117/D88289	*3ALb* (early solid)
Durum wheat	R *3ALb*	Pierce/PI 41353	*3ALb* (early solid)
Durum wheat	S *3ALa*	Pierce/PI 41353	*3ALa* (hollow)

Seeds were planted in a combination of MSU mix and Sunshine mix #1 in a 50:50 by volume ratio. MSU mix contained a composite of mineral soils from the Gallatin Valley, Canadian sphagnum peat moss and washed concrete sand in a 1:1:1 by volume ratio. Sunshine Mix #1 consists of a soil-less blend of Canadian Sphagnum peat moss and horticultural grade Perlite. All plants were watered daily and fertilized weekly using Peters Professional^®^ General Purpose Fertilizer (J.R. Peters, Inc., Allentown, Pennsylvania, United States) in aqueous solution of 100 ppm. Plants were grown in a greenhouse at the Plant Growth Center at Montana State University and maintained under greenhouse conditions (22°C ± 2°C day and 20°C ± 2°C night with a photoperiod of 15L:9D h). Plants were exposed to both natural and artificial light (GE Multivapor lamps; model MVR1000/C/U, GE Lighting, General Electric Co., Cleveland Ohio) and were rotated weekly to ensure uniform growth. Three plants from each line were chosen at the end of stem elongation, Zadoks stage 49, and at the completion of head emergence, Zadoks stage 59 (Zadoks et al., 1974). Stem tissue was collected and immediately frozen in liquid nitrogen and stored at 80°C until ready for further processing.

### 2.1 Plant processing for transcriptomics

Frozen wheat stems were ground to a fine powder in liquid nitrogen with a mortar and pestle. Stem powder was sent to Novogene (Novogene, CA, USA) for sample preparation and analysis. RNA was extracted using the TRIzol^®^ method. Briefly, 1 mL TRIzol^®^ was added to a microcentrifuge tube and homogenized and insoluble plant material was removed via centrifugation at 14,000 x g for 10 minutes at 4°C. The supernatant was transferred to an RNAse free microcentrifuge tube and chloroform was added at a ratio of 0.2mL chloroform to 1 mL TRIzol^®^. Samples were shaken vigorously and incubated at room temperature for 2-3 minutes before centrifugation at 14,000 x g for 15 minutes at 4°C. RNA was precipitated from the supernatant by addition of 0.5 mL isopropyl alcohol per 1 mL of TRIzol^®^. Samples were then incubated at room temperature for 10 minutes and centrifuged at 14,000 x g for 10 minutes at 4°C. Supernatant was removed and the RNA pellet was washed with 75% ethanol. RNA purity was checked using the NanoPhotometer^®^ spectrophotometer (IMPLEN, CA, USA). Integrity and quantitation of RNA samples were assessed using the RNA Nano 6000 Assay Kit of the Bioanalyzer 2100 system (Agilent Technologies, CA, USA).

### 2.2 Library preparation

NEBNext^®^ Ultra™ RNA Library Prep Kit for Illumina^®^ (NEB, USA) was used according to the manufacturer’s recommendations to create sequencing libraries. Sample mRNA was obtained from samples of total RNA using poly-T oligo-attached magnetic beads. The mRNA strands were fragmented using sonication with Diagenode bioruptor Pico. Reverse transcription was performed using random hexamer primer and M-MuLV Reverse Transcriptase (RNase H-) to obtain first strand cDNA. Both DNA Polymerase I and RNAse H were used for second strand cDNA synthesis. Introduction of exonuclease/polymerases ensured that remaining overhangs were converted to blunt ends. Adenylation was performed on the 3’ ends of resulting DNA fragments and NEBNext Adaptors with hairpin loop structure were ligated. Library fragments were purified with AMPure XP system (Beckman Coulter, Beverly, USA) to obtain only fragments of 150-200 bp. Selected cDNA was incubated with 3µL USER Enzyme (NEB, USA) at 37°C for 15 min followed by 5 min at 95°C before performing PCR with Phusion High-Fidelity DNA polymerase, PCR primers and Index (X) Primer. The resulting PCR products were purified using AMPure XP system and Agilent Bioanalyzer 2100 system was used to assess library quality. Clusters of the samples were generated using a cBot Cluster Generation System and PE Cluster Kit cBot-HS (Illumina) according to manufacturer instructions. Finally, libraries were sequenced on an Illumina platform to generate paired-end reads.

### 2.3 Data processing and analysis of transcriptomics data

Raw reads were processed through fastp to remove reads containing adapter sequences, poly-N sequences and low-quality reads. Resulting high-quality reads were used for all subsequent analyses. Reference genomes for spring wheat (IWGC RefSeq v1.0) and durum wheat (Svevo.v1) were downloaded from Ensembl release 104 (Howe et al., 2021). Reads were mapped to reference genomes using HISAT2 software. Mapped read numbers of each gene were counted using Featurecounts. Gene length and number of reads mapped to gene were used to calculate Reads Per Kilobase of exon model per Million mapped reads (RPKM). Differential expression analysis was performed using the DESeq2 R package to test for significant effects of allele, growth stage as well as any interactions between allele and growth stage (Love et al., 2014). P-values were adjusted for False Discovery Rate (FDR) using the approach of Benjamini and Hochberg (Benjamini and Hochberg, 2000). Differentially expressed genes were those with adjusted p-values <0.05 from a Wald test. Gene annotations and locations were identified using the EnsemblPlants database (http://plants.ensembl.org/Triticum_aestivum). Putative protein functions of annotated genes were characterized through the UniProt database (http://uniprot.org). Functional annotations of genes were obtained using the KEGG database (Kyoto Encyclopedia of Genes and Genomes, http://www.kegg.jp/). Since differences were observed between the parental and near isogenic lines containing the same allele, DESeq2 was also run with design formula ~group, where group was a combination of line and growth stage. This allowed identification of significant differences between samples containing the same allele using a Wald test.

Principal component analysis (PCA) of the 500 gene transcripts with the most variability was performed in R 3.4.1 using the plotPCA function of DESeq2, ggplot2 and ggfortify packages (Love et al., 2014; Wickham, 2016; Tang et al., 2016). The dataset was subjected to variance stabilizing transformation prior to PCA.

### 2.4 Plant processing for metabolomics

Frozen wheat stems were ground to a fine powder in liquid nitrogen with a mortar and pestle. Approximately 150 mg of stem powder was weighed and immersed in ice cold 100% methanol (MeOH). Samples were then vortexed and sonicated for 15 minutes at room temperature before centrifugation for 10 minutes at 25,000g. Proteins were separated from the supernatant by acetone precipitation with two and a half parts of acetone to one-part MeOH solution at -80°C overnight, followed by centrifugation at 4°C for 10 min. The resulting supernatant fraction was dried under nitrogen gas stream and stored at -80°C. Prior to analysis by liquid chromatography-mass spectrometry (LC-MS), samples were resuspended with 100 µL in 5:1 acetonitrile: water with 0.1% formic acid.

### 2.5 Liquid chromatography mass spectrometry

Metabolite analysis was conducted at the Montana State Mass Spectrometry Facility in Bozeman, Montana. Analysis was performed on a Waters Synapt G2 XS-XS Q-IMS-TOF interfaced with an i-class UPLC (Waters Corporation, MA, USA).

Metabolites were separated by normal-phase chromatography on an Acquity UPLC^®^ BEH HILIC 1.7 µm column (Waters Corporation, MA, USA) at 50°C with a flow rate of 400 µL min -1. The solvent program started with a two-minute step of 5% solvent A (0.1% formic acid in H2O; waste) with 95% solvent B (0.1% formic acid in acetonitrile) followed by a 95% to 50% solvent B gradient over 12 min, continued 50% solvent B for 2 min, and then a return to a 50% solvent A to 95% solvent B gradient over 5 minutes. Mass detection was performed in positive mode, with a scan range from 50 – 1200 m/z with scan time of 0.2 s. Capillary voltage was set to 2.53 V, sampling cone voltage was set to 40 V, and source offset voltage was set to 4 V. Cone gas temperature was 110°C with a flow rate of 30 L per hour -1. Desolvation temperature was 400°C with a flow rate of 500 L per hour. Nebulizer gas pressure was 3.5 bar. Leucine enkephalin, with a reference mass of m/z 556.2771, was used as the lock mass compound.

### 2.6 LC-MS data pre-processing and statistical analysis

Raw spectral data acquired in centroid mode was imported to Progenesis QI for pre-processing and compound identification (Waters Corporation, MA, USA). Each run in the experiment was compared to every other run and the run with the most similarity to all others was chosen as the reference run for automatic alignment. After review of the automatic alignment, the automatic sensitivity method was used to estimate noise levels in the data and perform peak picking across all runs. The resulting list of peaks was deconvoluted, meaning ions and adducts of the same compound were grouped together. Finally, compounds were identified using the Metacyc database, and resulting identifications were filtered manually to remove low quality peaks and low confidence identifications (Caspi et al., 2014).

Statistical analysis was performed using R version 4.1.3 using MetaboAnalystR (Pang et al., 2020). Log transformation and range scaling was used to meet the assumption of normality, and normalized values were used to perform a principal component analysis (PCA). A two-way ANOVA was conducted using line and growth stage as fixed effects to identify differences in metabolic profiles of the near isogenic lines and “parental” lines containing the same alleles at Zadoks stage 49 and Zadoks stage 59. Since differences were sometimes observed between parental lines and near isogenic lines with the same allele, the data was separated into a dataset containing only near isogenic lines and used to perform PCA as well as a two-way ANOVA using line and growth stage as fixed effects. *Post-hoc* testing was performed using Fisher’s least significant difference (LSD) test for multiple comparisons to identify significant differences between parental and near isogenic lines containing different alleles at Zadoks 49 and Zadoks 59. Data from the near isogenic line pairs was also separated into three individual datasets and used to perform the same analysis. Additionally, fold change analysis was performed to identify overall trends in metabolite expression.

## 3 Results

### 3.1 Principal component analysis of transcripts in spring wheat

**Figure 1:**
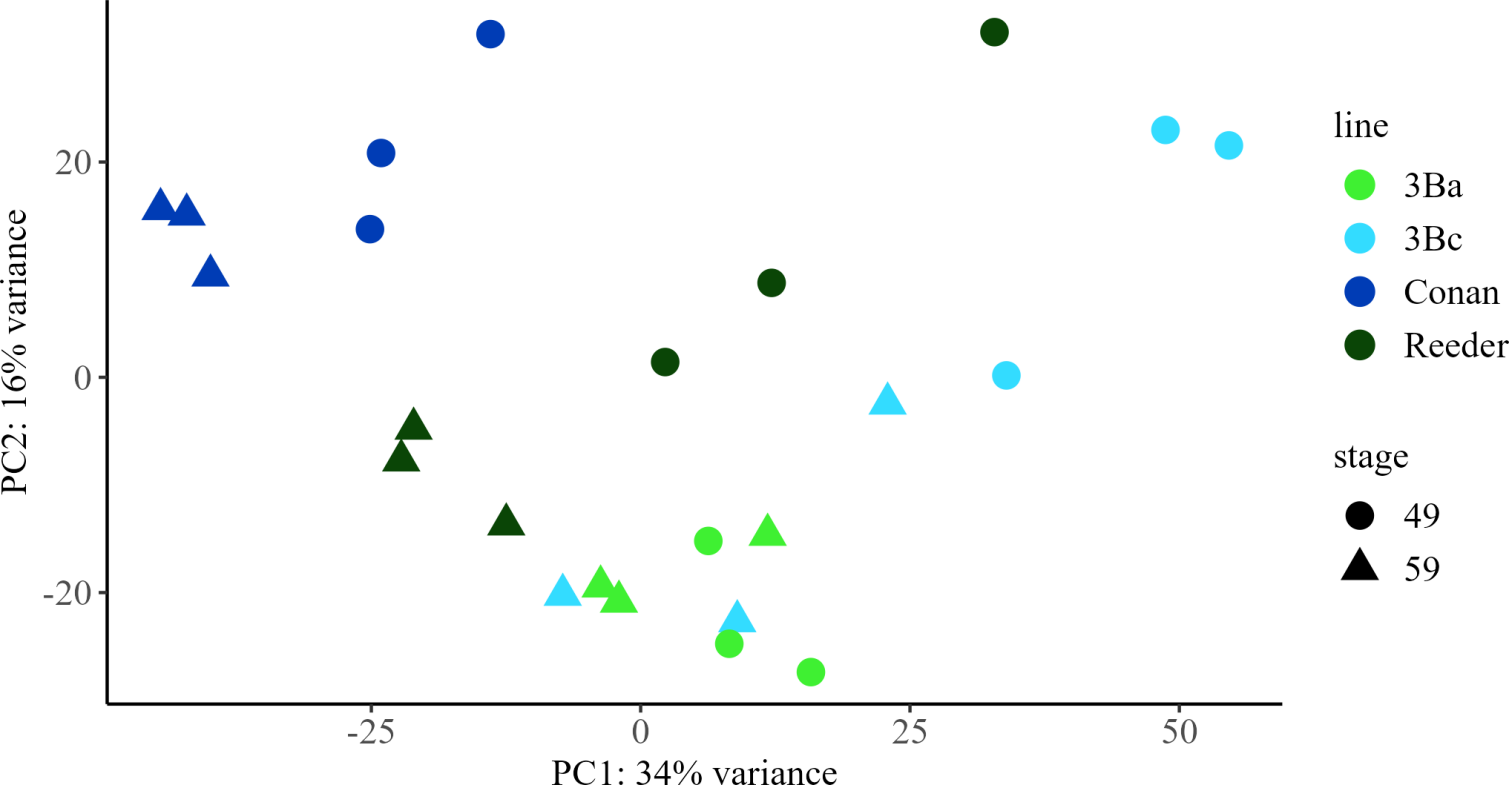
Principal component analysis (PCA) score plots for early (Zadoks 49) and late (Zadoks 59) stage samples of spring wheat transcripts from near isogenic lines with *3BLa* and *3BLc* alleles and parental lines Conan and Reeder. PCA plots were created using transcriptomic data from early and late stage plants with each point representing a sample from a main stem. *3BLa*, light green; *3BLc*, royal blue; Conan, dark blue; Reeder, dark green; early, circle; late, triangle.

A principal component analysis (PCA) was conducted on all 52,527 mapped genes (**Supplementary Tables 1**–**3**). The first principal component (PC1) explained 34% of the variance in the gene expression and showed clear segregation of the Conan and Reeder samples from the remaining samples of the near isogenic lines, while the second principal component (PC2) explained 16% of the variance in gene expression but was not able to discriminate between samples with the same allele or growth stage (**Figure 1**). Samples from NILs with the *3BLc* allele showed greater distribution than the samples with the *3BLa* allele.

### 3.2 Differential expression of transcripts in spring wheat

Of the 1,644 genes that were differentially expressed among NIL pairs, 118 were matched to chromosome 3B in the region of interest containing the microsatellite marker *Xgwm340* used for development of these NILs by Varella et al. (2015) (**Supplementary Table 4**). These genes had putative functions involving defense response, proteolysis, protein phosphorylation and transport and lignin biosynthesis.

*TraesCS3B02G458700* was identified as a cysteine-rich peptide CRP1, a type of antimicrobial peptide (AMP) with high stability. The number of transcripts was lower at Zadoks stage 59 for Reeder, Conan as well as in NILs with the *3BLa* and *3BLc* alleles, with the greatest decrease observed in NILs with the *3BLc* allele (**Supplementary Table 5**). In the Reeder samples and NILs with the *3BLa* allele, the relative abundance of this transcript differed slightly between growth stages, but this difference was not significant (**Supplementary Table 5**). In the Conan samples and NILs with the *3BLc* allele, the variability among samples was greater at Zadoks 49 (**Supplementary Figure 1A**). A 2-oxoglutarate and Fe(II)-dependent dioxygenase family gene, *TraesCS3B02G520100*, was significantly upregulated in Reeder and 3BLa samples at Zadoks 49 and Zadoks 59 (adjusted p-value=0.003, **Supplementary Figure 1B**). A possible lipoxygenase, *TraesCS3B02G602500*, was significantly upregulated in Conan and *3BLc* samples at both Zadoks 49 and Zadoks 59 (adjusted p-value=0.001, **Supplementary Figure 1C**). *TraesCS3B02G603900* was identified as *TaGATA38* by Cheng et al., 2021, based on the presence of a GATA zinc finger domain (Cheng et al., 2021). Abundance of this transcript was significantly upregulated in Reeder and *3BLa* samples at both growth stages (p-value <0.001, **Supplementary Figure 1D**).

*TraesCS3B02G601600*, a metallothionein, was identified as a candidate gene for the thickness of pith in a study of nitrogen use efficiency related traits in wheat (Zhao, 2019). Reeder samples had significantly increased abundance of this transcript at Zadoks 49 compared to Conan and *3BLc* samples, but *3BLa* samples showed much lower transcript abundance at this stage (p-value=0.039, **Supplementary Figure 1E**). Other candidate genes for stem diameter identified by Zhao (2019) were also differentially expressed between lines in this study, including *TraesCS3B02G608500*, an aquaporin; *TraesCS3B02G610100*, a pectin methylesterase; and *TraesCS3B02G612000*, a caffeic acid-oxy-methyltransferase. The putative aquaporin had significantly higher expression in Reeder samples at both growth stages compared to all others (adjusted p-value=0.021, **Supplementary Figure 1F**). However, Conan and *3BLc* samples also showed significant differences at Zadoks 49 (adjusted p-value=0.008, **Supplementary Table 1**). Similar expression patterns were observed for the putative pectin methylesterase (PME) and caffeic acid-oxy-methyltransferase (COMT) transcripts, both of which showed significantly higher abundance in the Reeder and *3BLa* samples (p-value=0.001, **Supplementary Figures 1G, H**). The putative pectin methylesterase (PME) also showed significant differences between Conan and *3BLc* NILs at Zadoks 59 (p-value=0.007, and also increased significantly with maturity in Conan plants (p-value=0.024, **Supplementary Table 1**).

3,272 genes were found to be differentially expressed between lines based on growth stage and 7 of these were located in the region of interest on chromosome 3B (**Supplementary Table 6**). Putative functions for these genes included regulation of transcription and transmembrane transport, though not all were fully annotated. *TraesCS3B02G596400*, *TraesCS3B02G597000*, and *TraesCS3B02G596800* are all members of the cytochrome p450 protein family, which is the largest family of enzymes in plants. *TraesCS3B02G596400* was significantly upregulated in early growth stages across all lines (p-value= 0.0009, **Supplementary Figure 2A**) which differed from the expression of the other two cytochrome p450 genes. *TraesCS3B02G597000* and *TraesCS3B02G596800* shared similar expression patterns, both were significantly upregulated in the NILs at early growth stages, and in Conan at later growth stages while no difference in expression between early and late growth stages was observed in Reeder (p-value=0.014 and 0.0267, **Supplementary Figures 2B, C** respectively). *TraesCS3B02G592500*, a PIN-LIKES protein, showed differential expression at all growth stages across all lines (p-value=0.0002, **Supplementary Figure 2D**). *TraesCS3B02G581900*, a putative uridylate kinase appeared to increase significantly at later growth stages but showed increased expression in Conan and near isogenic lines with the 3Bc allele, while Reeder and lines with the 3Ba allele showed little difference (p-value=0.022, **Supplementary Figure 2E**). *TraesCS3B02G583000* and *TraesCS3B02G597900*, identified as *TaVPE3cB* by Liu et al, 2023, have putative functions of protein binding and proteolysis respectively, and showed significant upregulation at later growth stages across all lines (p-value=0.035 and 0.007, **Supplementary Figures 2F, G** respectively).

Neither growth stage nor allele had a significant effect on expression of *TraesCS3B02G608800*, an ortholog of *SSt1* in durum wheat (p-value= 0.66, p-value=0.09, respectively, data not shown).

**Figure 2:**
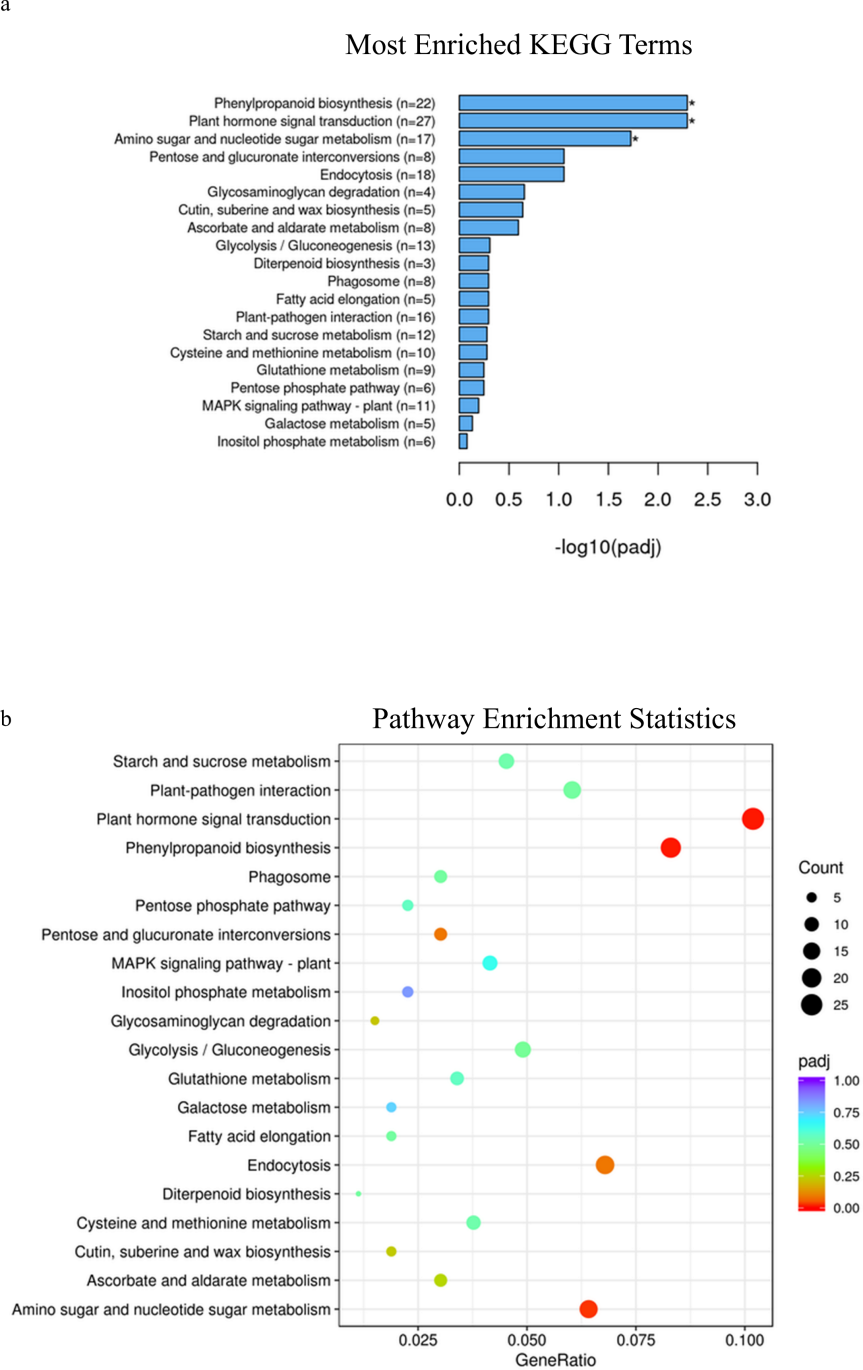
**(A)** KEGG enrichment histogram of the top 20 significantly enriched terms in KEGG enrichment analysis of spring wheat samples with the *3BLc* and *3BLa* alleles at Zadoks 49. **(B)** KEGG enrichment scatterplot of the top 20 significantly enriched terms in KEGG enrichment analysis of spring wheat samples with the *3BLc* and *3BLa* alleles at Zadoks 49.

In spring wheat at Zadoks stage 49, KEGG pathway enrichment analysis identified phenylpropanoid pathways, plant hormone signal transduction and amino sugar and nucleotide sugar metabolism as significantly enriched pathways associated with genes that were differentially expressed in samples with alleles for solid or early solid stems (adjusted p-value=.005, 0.005 and 0.01, **Figures 2A, B**; **Supplementary Table 7**), while at Zadoks 59 no pathways were found to be significantly associated with any of the differentially expressed genes (**Supplementary Figure 3A, B**; **Supplementary Table 8**). At Zadoks 49, enrichment of phenylpropanoid pathways were associated with upregulation of 19 genes in stems with the early solidness allele *3BLc*, including an aldehyde dehydrogenase, glycosyl hydrolase and an AMP-binding protein (**Supplementary Table 7**). There were 3 upregulated genes located on chromosome 3B, *TraesCS3B02G154200* and *TraesCS3B02G209900* with unknown annotations and *TraesCS3B02G426300*, another AMP binding protein (**Supplementary Table 7**). Enrichment of plant hormone signal transduction pathways was associated with downregulation of 14 genes in early solid stems including two genes on chromosome 3B with unknown annotations, *TraesCS3B02G127400* and *TraesCS3B02G380300*. Upregulation of 17 genes in early solid stems was associated with significant enrichment of amino sugar and nucleotide sugar metabolism pathways, but none of these genes were found on chromosome 3B (**Supplementary Table 7**). These included glycosyl hydrolases, glycotransferases, a phosphomannomutase/phosphoglucomutase (PMM/PGM) and a galacto-, homoserine-, mevalonate-, phosphomevalonate-kinase (GHMP kinase) (**Supplementary Table 7**).

### 3.3 Principal component analysis of metabolites in spring wheat

**Figure 3:**
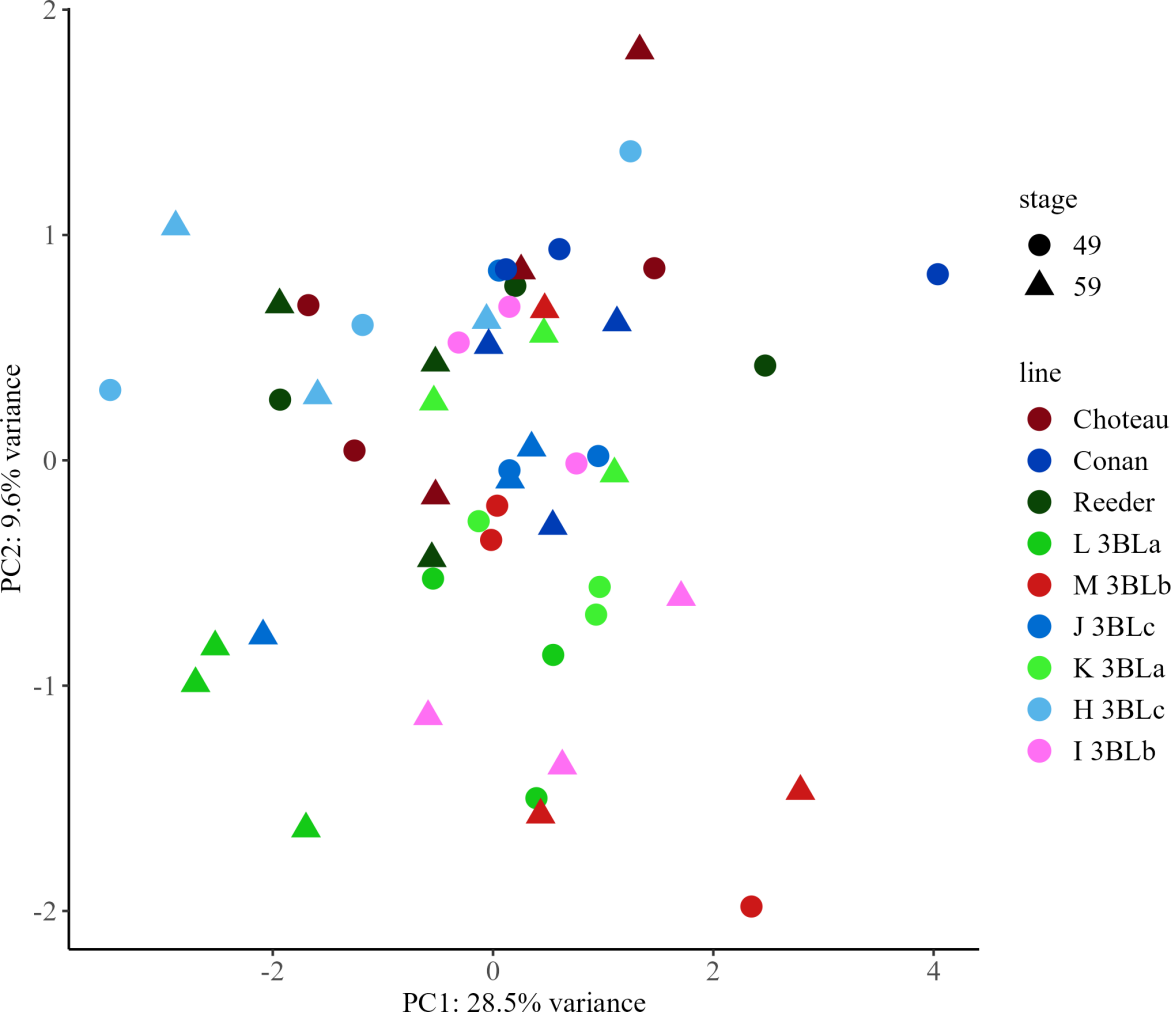
Principal Component Analysis (PCA) score plots for early (Zadoks 49) and late (Zadoks 59) stage samples of spring wheat metabolites from parental lines Choteau, Conan and Reeder and near isogenic lines with *3BLa*, *3BLb* and *3BLc* alleles. PCA plots were created using LC-MS data from early and late stage plants with each point representing a sample from a main stem. Choteau, maroon; Conan, dark blue; Reeder, dark green; L *3BLa*, green; M *3BLb*, red; J *3BLc*, royal blue; K *3BLa*, light green; H *3BLc*, light blue; I *3BLb*, pink; early, circle; late, triangle.

In spring wheat samples, there were 186 features, referred to here as metabolites (**Supplementary Table 9**). Of these, 35 compounds were identified (**Supplementary Table 10**). Using data from the parental lines as well as the near isogenic lines, principal components analysis (PCA) was performed on all 186 metabolites (**Supplementary Tables 1**–**3**). The first principal component (PC1), explained 28.5% of the variability in the dataset and the second principal component (PC2), explained 9.6% of the variability in the dataset. Neither PC1 nor PC2 was able to discriminate between groups of samples with the same allele or at the same growth stage (**Figure 3**).

### 3.4 Differential expression of metabolites in spring wheat

At Zadoks 49, fold change analysis identified 5 metabolites with increased abundance in fully solid stemmed lines compared to hollow stemmed lines, including 1-phosphatidyl-1D-myo-inositol 3-phosphate and santene hydrate (p-values<0.05, **Supplementary Table 11**). Twenty metabolites were found with increased abundance in lines with the early stem solidness allele when compared to hollow stemmed lines, including the compounds 1-phosphatidyl-1D-myo-inositol 3-phosphate, 4-(4-hydroxyphenyl)-2-butanone O-[2-galloyl-6-p-coumaroylglucoside], feruloyl-2-hydroxyputrescine and riboflavin (p-values<0.05, **Supplementary Table 11**). Six metabolites were also found to have increased abundance in early solid lines compared to solid stemmed lines at this growth stage (p-values<0.05, **Supplementary Table 11**). At Zadoks 59, 57 metabolites were identified which had increased abundance in solid stemmed lines compared to hollow stemmed lines, including fucose 1-phosphate, ethyl (S)-3-hydroxybutyrate glucoside and semilicoisoflavone B (p-values<0.05, **Supplementary Table 11**). Twelve metabolites were identified which had increased abundance in lines with the early stem solidness allele compared to hollow stemmed lines, including prunin 6’’-O-gallate (p-values<0.05, **Supplementary Table 11**). Twenty-four metabolites were also found with increased abundance in early solid lines compared to solid stemmed lines with the *3BLb* allele at Zadoks 59 (p-values<0.05, **Supplementary Table 11**).

**Figure 4:**
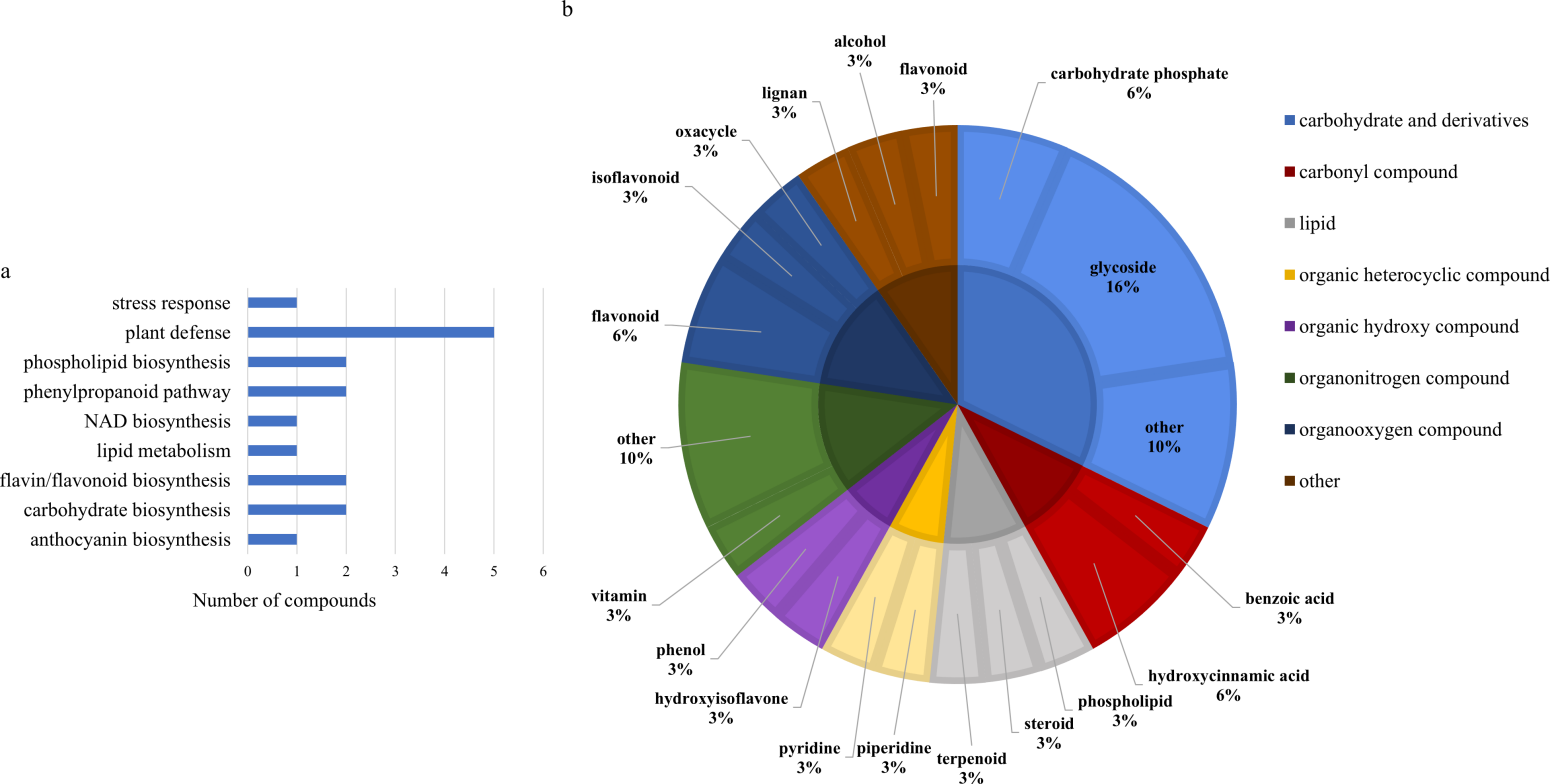
**(A)** Biochemical pathway associations of significant compounds in the spring wheat dataset which consisted of all samples from spring wheat parental and near isogenic lines. Significant compounds (p-values<0.05) were identified using two-way ANOVA. **(B)** Classifications of significant putatively identified metabolites in the spring wheat dataset. The innermost circle indicates the compound class with the outer ring representing compound subclass. Carbohydrate and derivatives, royal blue; carbonyl compound, red; lipid, grey; organic heterocyclic compound, yellow; organic hydroxy compound, purple; organonitrogen compound, dark green; organooxygen compound, dark blue; other, brown.

Two-way ANOVA of all identified metabolites from parental and near isogenic lines identified 35 metabolites which showed significant differences based on either line or growth stage (p-value<0.05, **Supplementary Tables 10**, **12**). There was evidence for an interaction between line and growth stage for 4-(4-hydroxyphenyl)-2-butanone O-[2-galloyl-6-p-coumaroylglucoside], 4-hydroxybenzeneacetonitrile, feruloyl-2-hydroxyputrescine, flavone, (-)-dioxibrassinin, kievitol, riboflavin, 3-hydroxy-4-butanolide, ethyl (S)-3-hydroxybutyrate glucoside and prunin 6’’-O-gallate (p-value<0.05). Growth stage had a significant effect on 16 metabolites, 1-O-caffeoyl-(b-D-glucose 6-O-sulfate), 3,5-digalloylepicatechin, 4-(4-hydroxyphenyl)-2-butanone O-[2-galloyl-6-p-coumaroylglucoside], caffeoylmalic acid, 1-phosphatidyl-1D-myo-inositol 3-phosphate, (+)-12a-hydroxypachyrrhizone, (-)-dioxibrassinin, sucrose, 2-O-galloylgalactaric acid and an unknown O-glycosyl compound based on growth stage (p-value<0.05). Significant differences were also observed in 26 metabolites based on line, including 2,2,6,6-tetramethyl-4-piperidinone, feruloyl-2-hydroxyputrescine, riboflavin, kievitol, flavone, cyclocalamin, 4-(4-hydroxyphenyl)-2-butanone O-[2-galloyl-6-p-coumaroylglucoside], (-)-dioxibrassinin, santene hydrate, 3,5-digalloylepicatechin, fucose 1-phosphate, 1-phosphatidyl-1D-myo-inositol 3-phosphate, glycerol 3-phosphate, 3-hydroxy-4-butanolide, dumetorine, cinncassiol D4, an unknown O-glycosyl compound, mevalonic acid-5P, 4-hydroxybenzeneacetonitrile, an unknown benzenoid, methyl 3-(2,3-dihydroxy-3-methylbutyl)-4-hydroxybenzoate, benzoyl glucuronide, ethyl (S)-3-hydroxybutyrate glucoside, N,N’-Bis(gamma-glutamyl)cystine, semilicoisoflavone B, 8-hydroxypinoresinol, cyanidin 3-rutinoside, 4’-methyl-(-)-epigallocatechin 3’-glucuronide, prunin 6’’-O-gallate and niacinamide (p-value<0.05). Of the 36 significantly different features, 30 were assigned to a class and 17 could be matched to their respective biological pathways. Most of the features were classified as carbohydrates or carbohydrate derivatives, followed by flavones, flavonoids and isoflavonoids. The majority of these metabolites were found to be directly involved in plant defense, followed by lipid/phospholipid metabolism, phenylpropanoid biosynthesis and metabolism, carbohydrate biosynthesis and metabolism and flavin/flavonoid biosynthesis (**Figure 4**).

### 3.5 Statistical analysis of spring wheat NIL data only

Principal components analysis of metabolites was performed using the near isogenic line dataset. The first principal component (PC1) explained 29.5% of the variability in the dataset and the second principal component (PC2) explained 10.8% of the variability in the dataset. As expected for a global metabolite data set, neither principal component was able to help define groups by allele or growth stage (**Supplementary Figure 4**). Two-way ANOVA using the near isogenic line dataset identified 29 metabolites which showed significant differences based on the specific line and growth stage (p-value<0.05, **Supplementary Table 13**). There was evidence for an interaction between line and growth stage for nine metabolites including flavone, feruloyl-2-hydroxyputrescine, 4-(4-hydroxyphenyl)-2-butanone O-[2-galloyl-6-p-coumaroylglucoside], 3-hydroxy-4-butanolide, 6-gingesulfonic acid, kievitol, riboflavin, (-)-dioxibrassinin and 4-hydroxybenzeneacetonitrile (p-value<0.05). Significant differences based on growth stage were observed in 1-O-caffeoyl-(b-D-glucose 6-O-sulfate), kievitol, 4-(4-hydroxyphenyl)-2-butanone O-[2-galloyl-6-p-coumaroylglucoside], 3,5-digalloylepicatechin, caffeoylmalic acid, 4’-methyl-(-)-epigallocatechin 3’-glucuronide, riboflavin, 8-hydroxypinoresinol, an unknown O-glycosyl compound, 2-O-galloylgalactaric acid, 6-gingesulfonic acid,(-)-dioxibrassinin, fucose 1-phosphate and 1-phosphatidyl-1D-myo-inositol 3-phosphate (p-value <0.05). Twenty metabolites were found to be significantly different based on line, these included feruloyl-2-hydroxyputrescine, flavone, 2,2,6,6-tetramethyl-4-piperidinone, kievitol, riboflavin, 4-(4-hydroxyphenyl)-2-butanone O-[2-galloyl-6-p-coumaroylglucoside], santene hydrate, (-)-dioxibrassinin, cyclocalamin, 4-hydroxybenzeneacetonitrile, 3-hydroxy-4-butanolide, a benzenoid, 3,5-digalloylepicatechin, 3,5,6-trihydroxy-3’,4’,7-trimethoxyflavone 3-glucuronide, benzoyl glucuronide, glabrin C, benzyl beta-primeveroside, dumetorine, castaneiolide and hirsutin (p-value<0.05). Of the 29 significantly different metabolites, 27 were assigned to a class and 10 were matched to their respective biological pathway (**Supplementary Figure 5**). The majority of these metabolites were classified as carbohydrates or carbohydrate derivatives as well as flavone, flavonoids or isoflavonoids and were involved in plant defense, flavin and flavonoid biosynthesis as well as the phenylpropanoid pathway.

### 3.6 Statistical analysis of NIL pair data

Using the metabolomics dataset from the NIL pair made up of lines containing the *3BLc* and *3BLb* alleles in a Vida background, principal components analysis (PCA) was performed. The first principal component (PC1) explained 36.9% of the variability in the dataset and was able to differentiate between growth stages in lines with the *3BLb* allele. The second principal component (PC2) explained 16.7% of the variability in the dataset. Lines with the *3BLc* allele showed greater spread, especially at the early growth stage (**Supplementary Figure 6**).

Two-way ANOVA identified six metabolites which showed significant differences between line and growth stage, with no evidence for an interaction for any metabolite (p-value<0.05, **Supplementary Table 14**). Significant differences based on allele were observed for (-)-dioxibrassinin, mevalonic acid-5P, 4-hydroxybenzeneacetonitrile, 3-hydroxy-4-butanolide, flavone, and feruloyl-2-hydroxyputrescine (p-value<0.05), while significant differences based on growth stage were also observed for 4-hydroxybenzeneacetonitrile, 3-hydroxy-4-butanolide, flavone and feruloyl-2-hydroxyputrescine.

PCA was also performed using the dataset from the NIL pair with lines containing the *3BLa* and *3BLb* alleles. The first principal component (PC1) explained 37.7% of the variability in the dataset and was able to distinguish between growth stages for lines with the *3BLa* allele. The second principal component (PC2) explained 15.4% of the variability in the dataset (**Supplementary Figure 7**).

Two-way ANOVA identified four metabolites that were significantly different based on the specific line and growth stage with no evidence of interaction (p-value<0.05). S-(hydroxymethyl) glutathione, citronellyl anthranilate, 6-gingesulfonic acid and 3,5,6-trihydroxy-3’,4’,7-trimethoxyflavone 3-glucuronide were all significantly different based on allele (p-value<0.05, **Supplementary Table 15**), with 6-gingesulfonic acid also showing a significant difference based on growth stage (p-value<0.05, **Supplementary Table 15**).

Finally, PCA was performed using the dataset from the NIL pair made up of lines containing the *3BLc* and *3BLa* alleles. The first principal component (PC1) explained 25.5% of the variability in the dataset and was able to separate *3BLa* lines by growth stage. The second principal component (PC2) explained 15.9% of the variability in the dataset. Two-way ANOVA did not identify any metabolites with significant differences in the *3BLc*/*3BLa* near isogenic line dataset (**Supplementary Figure 8**).

### 3.7 Principal component analysis of transcripts in durum wheat

**Figure 5:**
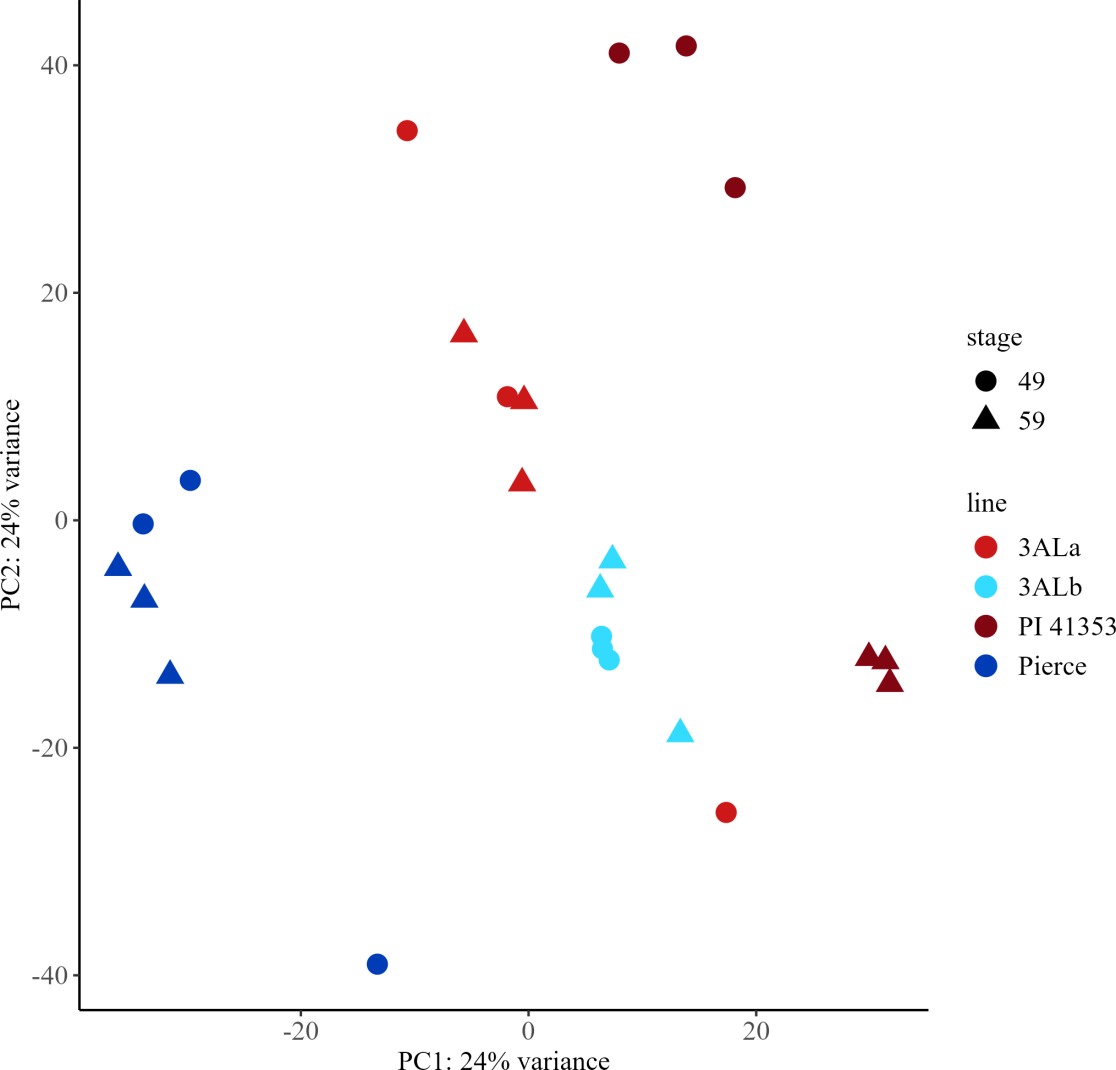
Principal Component Analysis (PCA) score plots for early (Zadoks 49) and late (Zadoks 59) stage samples of durum wheat transcripts from parental lines Pierce and PI 41353 and near isogenic lines with *3ALa* and *3ALb* alleles. PCA plots were created using transcriptomic data from early and late stage plants with each point representing a sample from a main stem. *3ALb*, light blue; *3ALa*, red; Pierce, dark blue; PI 41353, maroon; early, circle; late, triangle.

PCA was conducted on all 46,587 mapped durum wheat genes (**Supplementary Tables 16**–**18**). The first principal component explained 24% of the variance in the gene expression and showed clear segregation of the Pierce and PI 41353 samples from the remaining samples of the near isogenic lines, while the second principal component explained 24% of the variance but was unable to discriminate between samples of the same allele or growth stage (**Figure 5**). Samples with the *3ALa* allele showed greater spread than the samples with the *3ALb* allele.

### 3.8 Differential expression of transcripts in durum wheat

Of the 2,128 genes that were differentially expressed between lines based on allele, 146 were matched to chromosome 3A, although just three differentially expressed genes were located within the confidence interval of the marker positions in the Svevo genome used to derive the 3A NILs (chr3A: 514130564 to chr3A: 517062504) (Varella et al., 2019a; Avila et al., 2021) (**Supplementary Table 19**). These genes were *TRITD3Av1G185620*, an isoleucine-tRNA ligase, *TRITD3Av1G185730*, a senescence regulator with unknown domain and *TRITD3Av1G185910*, a MADS-box transcription factor. *TRITD3Av1G185620* and *TRITD3Av1G185910* were significantly upregulated in hollow stemmed susceptible plants PI 41353 and NILs with the *3ALa* allele (p-values 0.009 and 0.00038, **Supplementary Figures 9A, B** respectively).

In the durum wheat dataset, significant fold change differences were often observed between parental lines and NILs which shared the same allele (**Supplementary Table 20**). Additionally, six differentially expressed genes on chromosome 3A outside of the NIL region matched expression profiles in comparisons of the solid stemmed cultivar CDC Fortitude and the hollow stemmed pithless1 mutant utilized in the study by Nilsen et al, 2020. Genes that were upregulated in lines with the early stem solidness allele *3ALb* included *TRITD3Av1G174790*, a gene encoding for a FAM204A protein (p-value<0.0001, **Supplementary Figure 9C**), *TRITD3Bv1G198920*, a GDSL esterase/lipase (p-value=0.02, **Supplementary Figure 9D**), *TRITD4Av1G252660*, a glyceraldehyde 3-phosphate phosphatase gene (p-value=0.002, **Supplementary Figure 9E**), *TRITD6Av1G226950*, an alpha-1,3-mannosyl-glycoprotein 2-beta-N-acetylglucosaminyltransferase gene (p-value<0.0001, **Supplementary Figure 9F**) and *TRITD7Bv1G232630*, a putative cysteine protease (p-value=0.0003, **Supplementary Figure 9G**). The sixth gene that shared an expression pattern with CDC Fortitude and the pithless1 mutant was *TRITD7Bv1G015280*, a gene encoding for a 2-oxoglutarate (2OG) and Fe(II)-dependent oxygenase superfamily protein. This gene was significantly downregulated in lines with the early stem solidness allele *3ALb* (p-value=0.035, **Supplementary Figure 9H**). *TRITD4Av1G252660*, the putative glyceraldehyde 3-phosphate phosphatase gene, was also found to be differentially expressed between PI 41353 and NILs with the *3ALa* allele at both Zadoks 49 and 59 (p-value=0.036 and p-value<0.0001 respectively), and between Pierce and NILs with the *3ALb* allele at Zadok’s 59 (p-value=0.037).

Of the 3,206 genes that were differentially expressed between lines based on growth stage, 239 were matched to chromosome 3A, although just three differentially expressed genes were located within the region of interest (**Supplementary Table 21**). *TRITD3Av1G185730*, a senescence regulator protein with unknown functional domain and *TRITD3Av1G186090*, an ATP-binding cassette (ABC) transporter family protein were both significantly downregulated in lines with the *3ALb* allele (p-value=0.022 and <0.0001 respectively, **Supplementary Figures 10A, B**) while *TRITD3Av1G186350*, a malic enzyme was upregulated in these lines (p-value=<0.0001, **Supplementary Figure 10C**). *TRITD3Av1G186350* was differentially expressed between Zadoks 49 and 59 for *3ALa* NILs (p-value=0.016), PI 41353 (p-value=0.0004) and *3ALb* NILs (p-value=0.007). Differential expression was also observed between Pierce and *3ALb* lines at Zadoks 59 (p-value=0.021). *TRITD3Av1G186090* was also differentially expressed between growth stages in PI 41353 (p-value= 0.005). Outside of the 3A region, allele had a significant effect on the abundance of terpene synthase gene transcripts from chromosome 2B and 7B, *TRITD7Bv1G200640*, *TRITD2Bv1G006590*, *TRITD2Bv1G006640* and *TRITD2Bv1G051850* while growth stage had also had a significant effect on *TRITD7Bv1G200640* as well as *TRITD2Bv1G210370*.

*SSt1* (*TRITD3Bv1G280530*) was identified in both the parental and near isogenic lines, but neither allele nor growth stage had significant expression differences (p-value=0.987 and 0.948 respectively, **Supplementary Figure 9I**).

**Figure 6:**
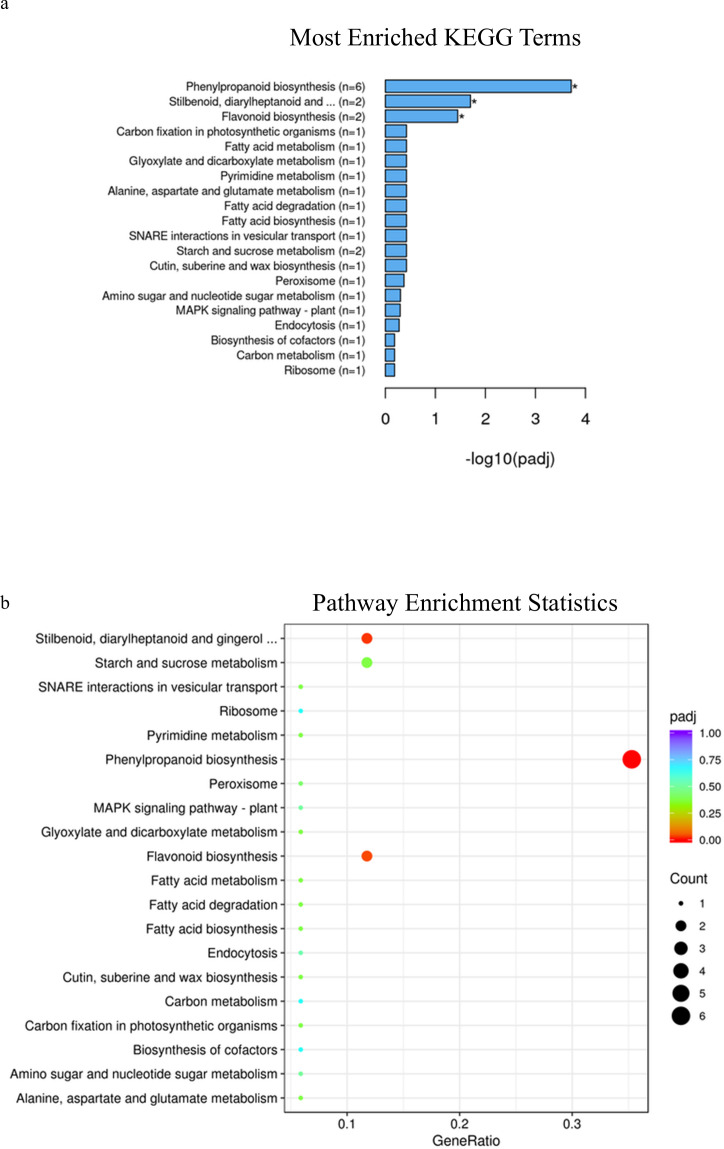
**(A)** KEGG enrichment histogram of the top 20 significantly enriched terms in KEGG enrichment analysis of durum wheat samples with the *3ALb* and *3ALa* alleles at Zadoks 49. **(B)** KEGG enrichment scatterplot of the top 20 significantly enriched terms in KEGG enrichment analysis of durum wheat samples with the *3ALb* and *3ALa* alleles at Zadoks 49.

**Figure 7:**
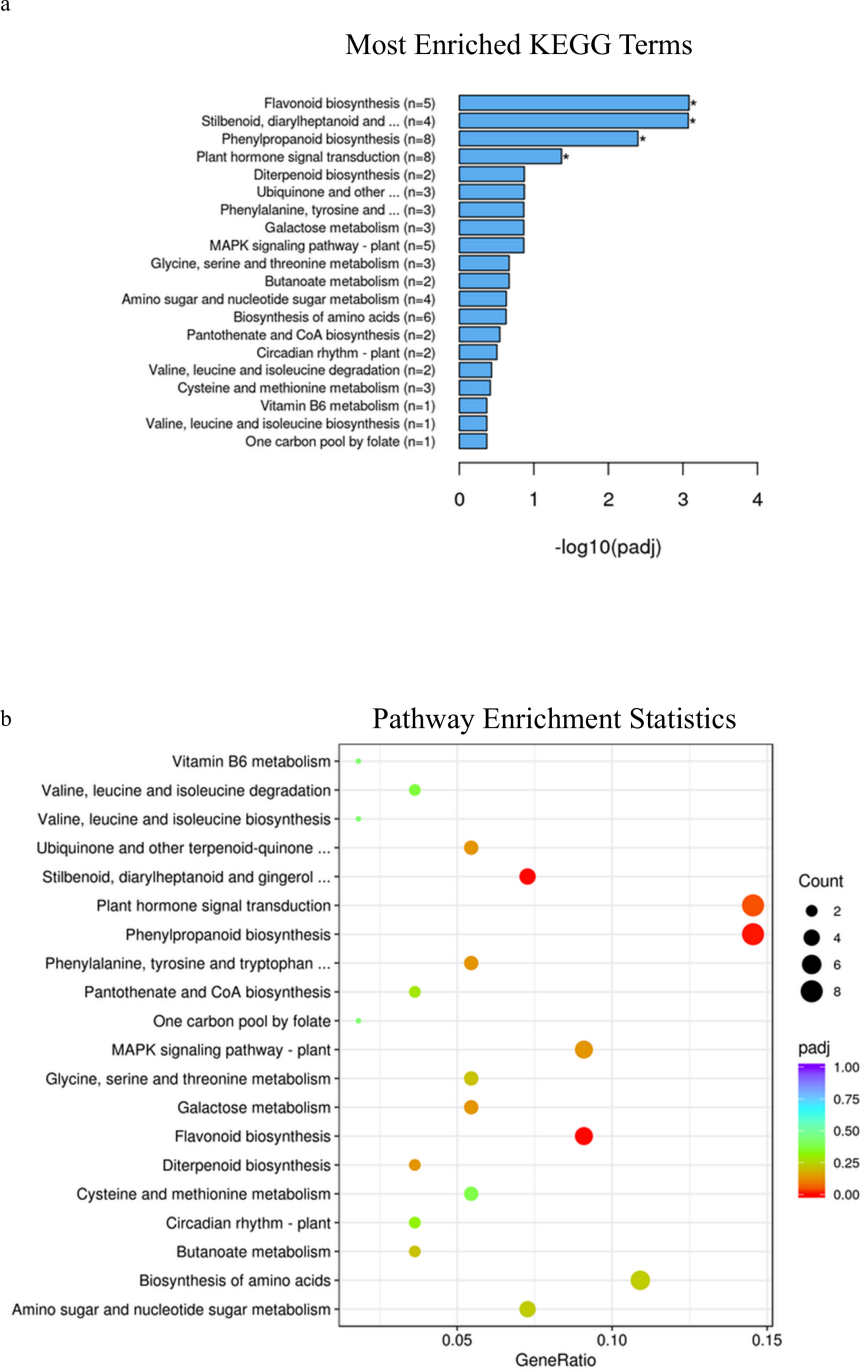
**(A)** KEGG enrichment histogram of the top 20 significantly enriched terms in KEGG enrichment analysis of durum wheat samples with the *3ALb* and *3ALa* alleles at Zadoks 59. **(B)** KEGG enrichment scatterplot of the top 20 significantly enriched terms in KEGG enrichment analysis of durum wheat samples with the *3ALb* and *3ALa* alleles at Zadoks 59.

At Zadoks stage 49, KEGG pathway enrichment analysis identified phenylpropanoid pathways, stilbenoid, diarylheptanoid and gingerol biosynthesis as well as flavonoid biosynthesis as significantly enriched pathways associated with differentially expressed genes in samples with different alleles in durum wheat (adjusted p-value=0.0001, 0.01 and 0.03 respectively, **Figures 6A, B**; **Supplementary Table 22**), while at Zadoks 59 these pathways were also found to be significantly associated with differentially expressed genes in addition to plant hormone and signal transduction pathways (adjusted p-value=0.004, 0.0008, .0008 and 0.04, **Figures 7A, B**; **Supplementary Table 23**). At Zadoks 49, enrichment of phenylpropanoid pathways were mostly associated with downregulation of 5 genes, none of which were located on chromosome 3A (**Supplementary Table 22**). These included 4 peroxidases and one putrescine hydroxycinnamoyltransferase (**Supplementary Table 22**). At Zadoks 59, enrichment of plant hormone signal transduction pathways was associated with downregulation of 5 genes including one gene on chromosome 3A (TRITD3Av1G033610, an auxin responsive protein)(**Supplementary Table 23**). At Zadoks 59, stilbenoid, diarylheptanoid and gingerol biosynthesis and flavonoid biosynthesis pathways were both significantly associated with downregulation of four genes each (**Supplementary Table 23**). Similar to results found at Zadoks 49, 8 differentially expressed genes associated with the phenylpropanoid pathway were downregulated at the later growth stage, including a peroxidase, trans-cinnamate 4-monooxygenase, caffeoyl-CoA O-methyltransferase, trans-caffeoyl-CoA 3-O-methyltransferase, cinnamoyl-CoA reductase and 2 genes with unknown annotations, although none were found on chromosome 3A (**Supplementary Table 23**).

### 3.9 Principal component analysis of metabolites in durum wheat

**Figure 8:**
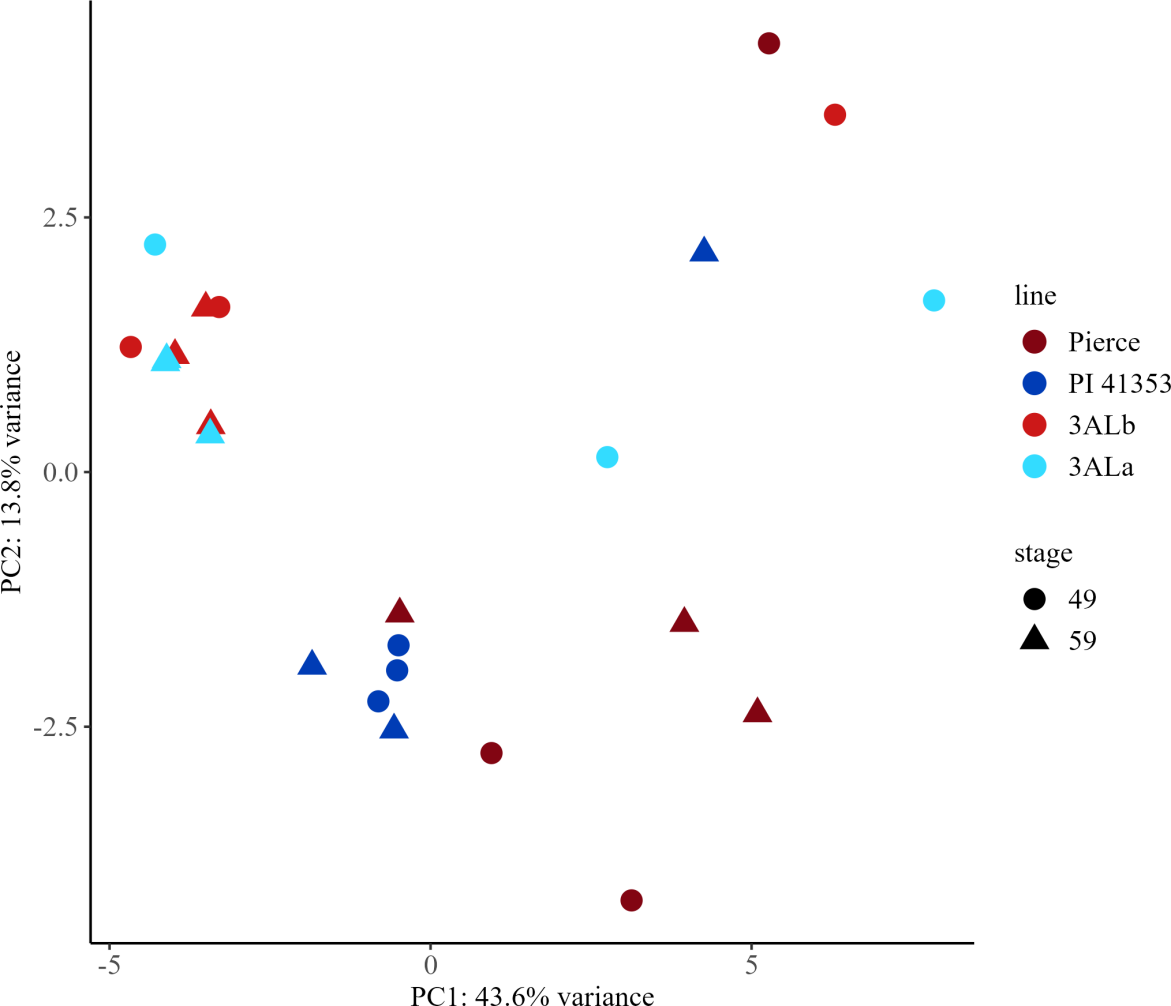
Principal component analysis (PCA) score plots for early (Zadoks 49) and late (Zadoks 59) stage samples of durum wheat metabolites from parental lines Pierce and PI 41353 and near isogenic lines with *3ALb* and *3ALa* alleles. PCA plots were created using LC-MS data from early and late stage plants with each point representing a sample from a main stem. Pierce, maroon; PI 41353, dark blue; *3ALb*, red; *3ALa*, light blue; early, circle; late, triangle.

In durum wheat samples, there were 346 features, referred to here as metabolites (**Supplementary Table 24**). Of these, 47 were compounds were identified (**Supplementary Table 25**). Principal components analysis was performed on all 346 identified metabolites (**Supplementary Tables 16**–**18**). The first principal component explained 43.6% of the variability in the dataset and was able to differentiate the parental lines from the near isogenic lines, while the second principal component explained 13.8% of the variability (**Figure 8**).

### 3.10 Differential expression of metabolites in durum wheat

Fold change analysis revealed 9 metabolites which had increased abundance in *3ALb* lines compared to *3ALa* lines at Zadoks 49, including 4-coumaroyl-2-hydroxyputrescine and DIBOA-Glc (p-values<0.05, **Supplementary Table 26**). Additionally, at Zadoks 59, 22 metabolites had increased abundance in *3ALb* lines compared to *3ALa* lines at the same growth stage, again including 4-coumaroyl-2-hydroxyputrescine and 2,4-dihydroxy-1,4-benzoxazin-3-one (DIBOA)-Glc (p-values<0.05, **Supplementary Table 26**).

**Figure 9:**
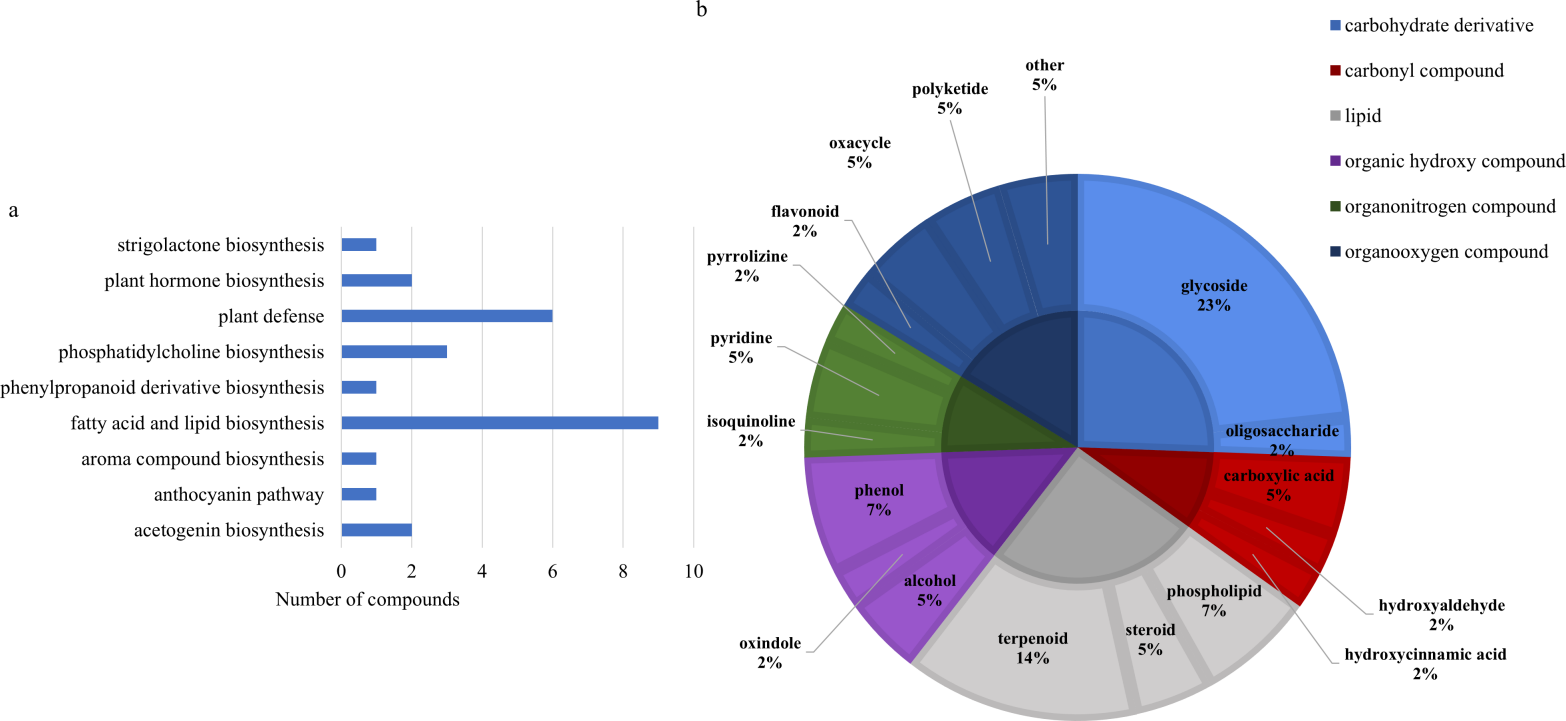
**(A)** Classifications of significant putatively identified metabolites in the spring wheat dataset. The innermost circle indicates the compound class with the outer ring representing compound subclass. Carbohydrate and derivatives, royal blue; carbonyl compound, red; lipid, grey; organic hydroxy compound, purple; organonitrogen compound, dark green; organooxygen compound, dark blue. **(B)** Biochemical pathway associations of significant compounds in the spring wheat dataset which consisted of all samples from spring wheat parental and near isogenic lines. Significant compounds (p-values<0.05) were identified using two-way ANOVA.

Two-way ANOVA of all data from parental and near isogenic lines of durum wheat revealed 48 metabolites that were significantly different based on stage or line (p-value<0.05, **Supplementary Tables 24**, **27**). There was evidence of an interaction for fourteen metabolites including cyanidin 3-rhamnoside, 2’-hydroxynicotine, lycopersiconol, 1-(2H-1,3-benzodioxol-5-yl)-2-[2,6-dimethoxy-4-(prop-2-en-1-yl)phenoxy]propyl acetate, canarigenin 3-[glucosyl-(1->4)-6-deoxy-alloside], laudanosine, 2’’,4’’,6’’-triacetylglycitin, sesaminol glucoside, PE-NMe(16:0/18:1(9Z)), an unknown annonaceous acetogenin, spinacoside D, ptelatoside B, neoreticulatacin A and cholesteryl-beta-D-glucoside. All 48 metabolites were significantly different only based on specific line (p-value<0.05), with the exception of cyanidin 3-rhamnoside which was significantly different based on line and growth stage (p-value<0.05). The majority of the significantly different metabolites were lipids or phospholipids, followed by glycosides, terpenoids and organooxygen compounds (**Figure 9**). These metabolites were mostly involved in fatty acid and lipid biosynthesis, phosphatidylcholine biosynthesis and plant defense (**Figure 9**).

For glucosides sesaminol glucoside, cassitoroside and taxifolin 3-arabinoside, lower variability between replicates was observed at the later growth stage. DIBOA-glucoside was generally lower in the samples from Zadoks 59 and was lower in lines with the resistant Pierce allele. Lipids cholesteryl-beta-D-glucoside and canarigenin 3-[glucosyl-(1->4)-6-deoxy-alloside] generally increased at later growth stages in all lines with the exception of Pierce. Lycopersiconol, the two unknown annonaceous acetogenins, and the three phospholipids showed opposite patterns of abundance in the near isogenic lines and their respective parents. 3,5-dihydroxy-6,7-didehydro-12’-apo-beta-caroten-12’-al, 28-Glucosyl-3b,23-dihydroxy-12,19(29)-ursadien-28-oate 3-arabinoside, spinacoside D and soyasaponin IV showed a decrease in abundance at Zadoks 59 in NILs containing the Pierce allele, while NILs containing the PI 41353 allele showed an increase at this growth stage. For these four metabolites, Pierce generally had the same abundance at both growth stages as NILs with the Pierce allele, but PI 41353 samples showed an opposite pattern to that of NILs with the PI 41353 allele. Furohyperforin and gibberellin A125 both had significant differences between both NIL pairs and the parental lines.

### 3.11 Statistical analysis of durum NIL data only

Principal components analysis was performed using only data from durum wheat near isogenic lines. The first principal component explained 57.3% of the variability in the dataset, while the second principal component explained 11.6% of the variability but neither component was able to differentiate the lines with the 3ALa allele from lines with the *3ALb* allele (**Supplementary Figure 11**).

A two-way ANOVA was performed using only data from near isogenic lines of durum wheat, but no metabolites with significant differences were observed (p-value>0.05).

## 4 Discussion

### 4.1 Phenylpropanoid pathway

The phenylpropanoid pathway is a complex pathway responsible for the biosynthesis of many secondary metabolites including benzenoids, cinnamates, flavonoids, tannins, lignans and lignins (Dixon et al., 2002; Vogt, 2010; Strack et al., 1987). Phenylpropanoids and their derivatives are involved in a wide range of processes including plant-environment interactions, plant defense, and formation of structural compounds such as lignin and suberin (Fraser and Chapple, 2011; Dixon et al., 2002). The phenylpropanoid pathway is proven to be upregulated in response to damage from insect pests such as Hessian fly and spruce bark beetle and is also induced upon fungal infection. This upregulation leads to formation of plant defense compounds as well as changes in lignin composition and cell wall fortification in leaves and stems of many species (Khajuria et al., 2013; Bi et al., 2010; Wainhouse et al., 1990). Most importantly, in recent omics studies of infested spring and winter wheat cultivars, the phenylpropanoid pathway has been implicated as part of the plant response to WSS infestation (Lavergne et al., 2020; Biyiklioglu et al., 2018).

In our complete spring wheat dataset, growth stage and allele had a significant effect on two compounds involved in phenylpropanoid biosynthesis, caffeoylmalic acid and feruloyl-2-hydroxyputrescine. Caffeoylmalic acid was found in higher abundance at the later growth stage in all NILs as well as all parental lines, while feruloyl-2-hydroxyputrescine had a variable change in abundance at the different growth stages depending on allele. Growth stage had a significant effect on the abundance of feruloyl-2-hydroxyputrescine while allele had a significant effect on the abundance of caffeoylmalic acid, with no evidence for an interaction between growth stage and allele for either compound. Allele also had a significant effect on abundance of feruloyl-2-hydroxyputrescine in the NIL only dataset, but in the NIL pairs datasets only the *3BLc*/*3BLb* set showed a significant effect of allele. There was also evidence of an interaction between allele and stage in the *3BLc*/*3BLb* set for feruloyl-2-hydroxyputrescine.

Hydroxycinnamic acid amides (HCAAs), including feruloyl-2-hydroxyputrescine, are accumulated in response to pathogen infection, and these compounds are often associated with cell wall thickening in resistant phenotypes (Samborski and Rohringer, 1970; Liu et al., 2018; Gunnaiah et al., 2012). Increased abundance of feruloyl-2-hydroxyputrescine in wheat rachis is also involved in resistance of wheat to *F. graminearum* through the same mechanism of cell wall reinforcement (Kosmolak, 1973). Feruloyl-2-hydroxyputrescine is induced in damaged tissues and also accumulates in systemic tissues in response to herbivory (Rivero et al., 2021). Caffeoylmalic acid also increases when plants are wounded, inducing a necrotic reaction with elicitation by salicylic acid (SA) (Housti et al., 2002). Our results show that cultivars resistant to WSS do not have higher abundance of HCAAs than susceptible cultivars, but it is possible that accumulation of these compounds could occur upon infestation with WSS, inducing cell wall changes that help defend against infestation or damage from feeding larvae.

In addition to small molecules related to phenylpropanoid metabolism, cultivar and growth stage both had significant effects on the abundance of transcripts of three genes related to lignin or pectin biosynthesis and organization in plant cell walls. *TraesCS3B02G612000*, a caffeic acid-oxy-methyltransferase (COMT) involved in lignin biosynthesis was upregulated in Reeder at the early growth stage and in lines with the *3BLa* allele (**Supplementary Figure 1H**). *TraesCS3B02G610100*, a pectin methylesterase (PME) was upregulated in Reeder and in lines with the *3BLa* allele, with lower abundance of transcripts at the later growth stage (**Supplementary Figure 1G**). *TraesCS3B02G596400*, a gene coding for a cytochrome p450 (CYP450) protein was significantly upregulated at the early growth stage in all lines, while other members of this protein family, *TraesCS3B02G597000* and *TraesCS3B02G596800*, were upregulated at the early growth stage in the near isogenic lines and in Conan at the late growth stage (**Supplementary Figures 2A–C**).

Depending on the species and location in the plant, COMT genes have various effects on total lignin and can alter the structure and proportion of lignin subunits in plant tissues (Baucher et al., 2003). In wheat, COMTs have been found to be associated with the S-lignin pathway and increased lignin content in leaves and heads (Bi et al., 2010; Li et al., 2021a). Generally, higher lignin content is associated with decreased herbivory due to physical hindrance and decreased palatability and nutrition (Santiago et al., 2013). While the cultivars in this study have not been screened for lignin content, selected hollow and solid stemmed cultivars have been used for past comparisons, and no differences were observed (McNeal et al., 1965). Despite the similarities in lignin content between solid and hollow stemmed cultivars, differential expression of these genes indicates that the Reeder allele may still cause changes in stem architecture that influence WSS behavior. This possibility is further supported by the results of an RNA-seq study by Oiestad et al. in 2017, in which upregulation of a putative O-methyltransferase with a COMT domain was upregulated in hollow stemmed cultivars (Oiestad et al., 2017). In Arabidopsis, COMT is also weakly induced when exposed to (Z)-3-hexenol (Yoneya and Takabayashi, 2014). In planta, (Z)-3-hexenol is converted to (Z)-3-hexenyl acetate, a compound identified as a potential semiochemical in the interaction between WSS and wheat (Matsui et al., 2012; Weaver et al., 2009). Reeder has been shown to release greater amounts of (Z)-3-hexenyl acetate than Conan so increased transcript abundance of COMT may be related to this observation (Weaver et al., 2009).

In wheat, PMEs are known to play a role in resistance to *Fusarium* head blight and stem rust (*Puccinia graminis f.* sp. *tritici*) by influencing the pattern of pectin methylesterification in plant tissues (Lionetti et al., 2012; Wiethölter et al., 2003). PMEs are critical to cell wall accessibility of pectin and are known to affect plant susceptibility to pests and pathogens in several species. In Arabidopsis, a significant decrease in aphid infestation was observed in plants where PME activity was inhibited (Silva-Sanzana et al., 2019). In leaf tissue of wheat lines susceptible to stem rust, methyl esters of homogalacturonans are distributed in a blockwise fashion instead of randomly due to the action of PMEs, this structure may assist in depolymerizing and inactivating enzymes produced by invading fungi (Wiethölter et al., 2003). In several studies, high mortality of WSS larvae was observed in wheat infected by *Fusarium* spp. either through direct infection of the larvae themselves or by an unknown pathogen-induced plant response (Wenda-Piesik et al., 2009; Piesik et al., 2009). Increased abundance of the PME gene in Reeder indicates that there may be differences in the structure of stem tissues which in turn may be responsible for the increased larval mortality in Fusarium infested plants as well as the greater incidence of WSS infestation in Reeder.

Cytochrome p450s (CYPs) are involved in a diverse array of reactions including those relating to plant defense, metabolism of fatty acids and biosynthesis of antioxidants and secondary metabolites (Pandian et al., 2020). A cytochrome p450 family gene, CYP707A1 is responsible for converting abscisic acid (ABA) to caffeoylmalic acid/phaseolic acid in Arabidopsis roots (Geng et al., 2013). An abscisic acid alcohol was identified in our dataset, and while growth stage and allele had no significant impact on abundance, a slight decrease at later growth stages was observed for Reeder and NILs with the *3BLa* allele. CYPs in the CYP98A subfamily are involved in monolignol biosynthesis and biosynthesis of other secondary metabolites in the phenylpropanoid pathway (Schoch et al., 2006; Sullivan and Zarnowski, 2010). The function of the cytochrome p450 genes identified here has not been determined, but transcripts for these genes were generally higher at early growth stages, while an increase in caffeoylmalic acid was observed at late growth stages. Our results indicate that ABA may be converted to caffeoylmalic acid as the plant matures, especially in Reeder and plants with the *3BLa* allele.

In addition to the specific compounds and gene transcripts found in spring wheat that were differentially expressed between samples with different alleles at Zadoks stage 49 and Zadok stage 59, KEGG enrichment analysis revealed the association of the phenylpropanoid pathway with several additional genes, including two AMP binding proteins, one of which was found on chromosome 3B. Proteins with an AMP binding domain are found in many plant species and have a wide range of functions, including development of florets, programmed cell death and lignin biosynthesis (Soltani et al., 2006; Liu et al., 2017). Enrichment of phenylpropanoid pathways was also associated with downregulation of genes in hollow stemmed lines of durum wheat at both Zadoks stage 49 and Zadoks stage 59, including several peroxidase and transferase genes found outside of chromosome 3A.

Other studies involving WSS resistance and plant response to WSS infestation have identified other compounds and proteins in the phenylpropanoid pathway. In an omics study of WSS infestation in Scholar and Choteau, a decrease in lignoceric acid and the protein phenylalanine ammonia-lyase was observed, indicating that plants infested with WSS larvae may experience decreased lignin formation (Biyiklioglu et al., 2018). Nilsen et al. found that hollow- and solid-stemmed lines of durum wheat showed differences in the phenylpropanoids 4-hydroxycinnamate, ferulate and coumaroylquinate which were generally higher at later growth stages with variable response depending on genotype (Nilsen et al., 2020). In spring and winter wheat, Lavergne et al., 2020 identified differential expression of phenylpropanoids and lignans in plants infested by WSS as well as several proteins related to the phenylpropanoid pathway, but plant response varied considerably depending on cultivar (Lavergne et al., 2020). The differing methods used across these publications limit making direct comparisons, but these results clearly show that the phenylpropanoid pathway changes in response to WSS infestation and that the response is highly variable and may depend on cultivar, growth stage, level of damage to the plant and stage of larval development.

### 4.2 Carbohydrates/sugars biosynthesis and glycosides

Carbohydrate metabolism in plants is complex, adaptable and highly regulated. Products of carbohydrate metabolism such as sugar and starch are important sources of energy and also serve as raw material for the biosynthesis of a wide range of organic compounds. In grain crops, carbohydrates are stored in the pith of stems during early growth and development and can be reallocated to developing heads during grain fill, increasing yield and providing the plant with protection against drought and cold stress (Blum, 1998; Saint Pierre et al., 2010). With discoveries of sugar-induced resistance genes, evidence has accumulated showing that sugars play a role as signaling molecules in response to wounding events, infection by pathogens and feeding by insect herbivores (Ahn et al., 1996; Berger et al., 1995; Schluepmann et al., 2004). Solid stem pith in spring and durum wheat is positively correlated with increased water-soluble carbohydrate (WSC) content which confers the plant with resistance to yield loss under drought conditions (Saint Pierre et al., 2010; Nilsen et al., 2020). While carbohydrate content varies considerably based on growth stage, internode and cultivar in our results and in several other studies, sugars and proteins involved in glycolysis and starch biosynthesis have also been implicated as part of the plant response to WSS infestation (Biyiklioglu et al., 2018; Lavergne et al., 2020). Additionally, it is known that WSS larvae feeding within the stem consume parenchyma and vascular tissues, and carbohydrate-rich parenchyma tissue makes up the majority of matter observed in the gut of feeding larvae (Ford et al., 1979; Holmes, 1949). Enzymes such as amylase, sucrase and cellulase are also present in the gut of WSS larvae, indicating that carbohydrates are hydrolyzed by the insect (Holmes, 1949).

In our complete spring wheat dataset, growth stage had a significant effect on the abundance of sucrose and several saccharide-related glycosides. Sucrose in stem tissue was higher at later growth stages in all parental lines and most of the NIL pairs. Near isogenic line pairs which did not show an increase in sucrose at the later growth stage showed no change between the early and late growth stages. Growth stage and line both had a significant effect on the abundance of fucose 1-phosphate, which decreased with maturity in all near isogenic lines regardless of allele. In the complete durum wheat dataset, line had a significant effect on several glycosides and oligosaccharides, while growth stage only had a significant effect on cyanidin 3-rhamnoside in the parental lines. Significant differences in glycosides in the durum wheat dataset were mostly due to differences between the NIL pairs and the parental lines which often showed opposite responses. For example, taxifolin 3-arabinoside increased at the later growth stage in the NILs but decreased slightly in the parental lines. KEGG enrichment analysis of the spring wheat transcriptome revealed upregulation of several genes associated with amino sugar and nucleotide sugar metabolism in stems with early stem solidness. Many of these genes were in the glycosyl hydrolase family, which are associated with changes in cell wall composition as well as development and elongation of roots and shoots (Banasiak et al., 2014; Nazipova et al., 2022).

While carbohydrate concentrations were variable depending on growth stage, line and specific compound in both the spring and durum wheat datasets, they may still be involved in host plant resistance by influencing the behavior of foraging females or affecting larval performance after ingestion. Carbohydrates are an important part of insect diets regardless of species, and concentration can have variable effects depending on species. Larvae of eastern spruce budworm, *Choristoneura fumiferana*, and spruce sawfly *Gilpinia hercyniae* show preference for and perform better on a carbohydrate rich diet (Albert and Jerrett, 1981; Jensen, 1988). Positive correlations have also been observed between carbohydrate levels in maize leaves and oviposition by European corn borer, *Ostrinia nubilalis* Hübner (Derridj et al., 1986, 1989). Some insect species do not perform as well when consuming a high carbohydrate diet. For example, tobacco hornworm larvae, *Manduca sexta* grew faster and had increased body size when feeding on leaves and artificial diets with lower concentrations of glucose and fructose (Machado et al., 2015). In addition to overall carbohydrate concentration, protein:carbohydrate (P:C) ratio also has an effect on both larval performance and the behavior of adult insects. Larvae of tobacco budworm, *Heliothis virescence*, grew more slowly on artificial diets with low P:C ratio and led to decreased performance of female pupae and lower egg production in adult females (Roeder et al., 2014). Adult *Drosophila* (*D. suzukii*) also prefer to oviposit on fruits and media with a low P:C ratio (Silva-Soares et al., 2017; Young et al., 2018). There is also some evidence that P:C ratio can affect the efficacy of secondary metabolites in plant resistance to insect pests (Machado et al., 2015; Perkovich and Ward, 2020; Raubenheimer, 1992). Adult WSS parasitoids *B. cephi* and *B. lissogaster* live longer and have increased egg load and egg volume when consuming carbohydrate-rich diets (Cavallini et al., 2022; Reis et al., 2019). It is possible that larvae of these parasitoid species also benefit from consuming sawfly larvae that have been feeding on plant tissue rich in carbohydrates, and continue to experience benefits after becoming adults.

Other studies involving the WSS system have identified metabolites and proteins associated with glycolysis and carbohydrate biosynthesis as part of the plant response to infestation. Lavergne et al. identified a pfkB-like carbohydrate kinase and a glyceraldehyde-3-phosphate dehydrogenase, two enzymes involved in starch biosynthesis and glycolysis respectively, which were upregulated in response to WSS infestation in the mildly resistant winter wheat cultivar Hatcher. They also found that in Conan, WSS infestation caused an increase in several enzymes involved in protein biosynthesis and proteolysis along with enzymes related to carbohydrate biosynthesis, including sucrose synthase (Lavergne et al., 2020). These results indicate that WSS infestation induces differences in total protein and carbohydrate concentration which seems to be related to plant resistance to WSS, but it is unclear whether these changes have an effect on the overall protein:carbohydrate ratio of the stem pith tissue or what effect this has on larval development. Another study of spring wheat cultivars also found that glyceraldehyde-3-phosphate dehydrogenase 1 was upregulated in the cultivars Choteau and Scholar after infestation with WSS as well as 3-phosphoglycerate a metabolite which is also involved in glycolysis (Biyiklioglu et al., 2018). In our durum wheat dataset a glyceraldehyde 3-phosphate phosphatase was upregulated in lines with the early stem solidness allele *3ALb*, although this gene is found outside the region of interest on chromosome 3A. Glyceraldehyde 3-phosphatase is not only a key component of glycolysis and glycerolipid biosynthesis, but evidence has also pointed towards its role in systemic acquired resistance (Mandal et al., 2011). The increased abundance of stem carbohydrates in solid or early solid stemmed cultivars as well as the induction of carbohydrate biosynthesis related pathways in infested plants further supports the suggestion that WSS infestation and possibly resistance are related to an overall increased energy demand by the plant, especially in resistant cultivars.

In several cereal crops, differences in WSC are also associated with changes in stem solidness, plant developmental stage and specific internode which are all factors that influence the oviposition behavior of WSS females (Nagata et al., 2002; Ford et al., 1979; Saint Pierre et al., 2010; Thevenot et al., 2005; Teulat et al., 2001). WSC are found in pith tissue with content of these carbohydrates higher in lower internodes compared to the peduncle or upper internode (Robinson et al., 2007; Li et al., 2015). Since these carbohydrates are found in the pith, concentrations are often higher in solid stemmed cultivars, a phenomenon also observed in the spring wheat cultivars used in this study (Ford et al., 1979; Nilsen et al., 2020). In both hollow and solid stemmed wheat cultivars, stem carbohydrates rapidly increase until several days after anthesis, when concentration declines (Ford et al., 1979). Female WSS choose to oviposit in the lowest internodes available that are actively elongating, where carbohydrate content is likely higher. They also commonly lay eggs in solid stem cultivars where larvae do not perform as well, but which do have increased WSC content compared to hollow stemmed cultivars, which suggests that WSC and other carbohydrates in the stem may be related to host plant selection and host plant resistance (Holmes and Peterson, 1960).

### 4.3 Lipids/phospholipids

Plant lipids are structurally diverse and therefore play many roles in the plant system in addition to energy and carbon storage. In plants, lipids act as structural components, signaling molecules, and precursors to compounds such as jasmonic acid that are involved in plant defense (Suh et al., 2022; Lavell and Benning, 2019; Reymond and Farmer, 1998). Phospholipids, galactolipids and sphingolipids are components of cell membranes that can sometimes act as signaling molecules under stress conditions (Laxalt and Munnik, 2002; Munnik et al., 1998). Lipids are also distributed in the thylakoid membrane and play a functional role in the assembly of photosynthetic complexes PSI and PSII (Yoshihara and Kobayashi, 2022).

Not only are lipids essential to plant growth and development, their involvement in host plant resistance and plant-insect interactions has also been well studied. Lipids are required by both plants and many insects that feed on them, and lipids and genes related to lipid biosynthesis and degradation change in response to insect infestation in many plants, although these changes are often highly variable depending on plant and insect species as well as lipid class (Zhu et al., 2012; Khajuria et al., 2013; Zhou et al., 2011; Kosma et al., 2010; Marti et al., 2013). Lipids are necessary for the normal growth and development of many insect species and are often acquired through feeding on plant material (Turunen, 1979). There are many insect species that store lipids prior to diapause, and these lipid stores are used throughout the overwintering period and are associated with increased larval survival (Sinclair and Marshall, 2018; Lehmann et al., 2020). Conversely, high concentrations of lipids in larval diets can also slow larval growth and even cause mortality in some species (Li et al., 2014; Liao et al., 2021).

In our complete spring wheat dataset, both line and growth stage had a significant effect on the abundance of 1-phosphatidyl-1D-myo-inositol 3-phosphate, a compound involved in sphingolipid biosynthesis which increased at the later growth stage in all lines, regardless of allele. Additionally, glycerol-3-phosphate, a molecule involved in glycolysis and the first step of glycerolipid biosynthesis, decreased significantly at the later growth stage.

In the complete durum dataset, allele had a significant effect on the phospholipids PE-NMe(16:0/18:1(9Z)) and PE-NMe(16:0/16:0). In both parental lines and NILs containing the Pierce allele, abundance of PE-NMe(16:0/18:1(9Z)) and PE-NMe(16:0/16:0) decreased at the later growth stage, while in NILs containing the PI 41353 allele, these phospholipids increased with maturity. *TRITD4Av1G252660*, a glyceraldehyde 3-phosphate phosphatase gene was also significantly upregulated in lines with the early stem solidness allele *3ALb* (**Supplementary Figure 9E**).

Other studies investigating stem solidness, the WSS tritrophic system and specifically plant response to WSS infestation have also identified differences in lipids, phospholipids or lipid-like compounds between cultivars. In comparisons of a *pithless* mutant and solid stemmed cultivar CDC Fortitude higher amounts of several lipids were identified in CDC Fortitude (Nilsen et al., 2020). Glycerol-3-phosphate also decreased with maturity regardless of cultivar (Nilsen et al., 2020). A study using infested spring and winter wheat showed that in the resistant cultivar Conan and mildly-resistant Hatcher there was an increase in some lipids and lipid-like molecules after WSS infestation, while the opposite effect was observed for other lipids (Lavergne et al., 2020). This variability in lipids across cultivars and in response to WSS infestation is not unique among plant-insect systems. In wheat isogenic lines resistant to the stem gall-forming Hessian fly, the total concentration of many lipids decreased during incompatible interactions while susceptible plants showed a slower and less marked decrease (Zhu et al., 2012). The opposite response is observed in maize, where phospholipids and several free fatty acids increased in response to Egyptian cotton leaf worm, *Spodoptera littoralis* (Boisd.) (Marti et al., 2013). Membrane lipids were reduced in resistant wheat infested with Hessian fly while free fatty acids and several intermediates in the phospholipid pathway increased, including glycerol-3-phosphate, an inducer of systemic acquired resistance (Khajuria et al., 2013; Chanda et al., 2011). Genes involved in lipid metabolism were upregulated while others were downregulated in rice infested with rice stem borer, *Chilo suppressalis* (Walker) a few days after introduction of the pest (Zhou et al., 2011). The results from these studies illustrate the variable nature of lipids related to insect resistant phenotypes and the need for further study of specific lipid response to WSS infestation.

### 4.4 Other spring wheat genes

Abundance of *TraesCS3B02G603900* (*TaGATA38*), was significantly upregulated in Reeder and *3BLa* lines at both growth stages, while Conan and lines with the *3BLc* allele showed no difference (**Supplementary Figure 1D**). In Arabidopsis, orthologs of *TaGATA38* are associated with seed germination, flowering and response to cold stress (Richter et al., 2013). An ortholog of *TaGATA38* in Brassica napus also had lower expression under drought and cold stress (Zhu et al., 2020). GATA genes in *Brachypodium* were also found to be sensitive to treatment with methyl jasmonate and salicylic acid and overexpression of *BdGATA13* in *Arabidopsis* increased chlorophyll content in leaves (Peng et al., 2021; Guo et al., 2021). In wheat, more than 75 GATA genes have been identified and classified into at least four subfamilies (Cheng et al., 2021; Feng et al., 2022). GATA genes in wheat show high diversity in expression under abiotic stresses depending on plant tissue, but *TaGATA38* has been found to have reduced expression in response to cold stress and phosphorus starvation and increased expression in response to heat and drought stress (Feng et al., 2022). While it has been proposed that GATA genes are responsible for regulating downstream genes involved in cold and drought stress, the exact mechanisms for the observed resistance to abiotic stresses have not yet been elucidated so it is unclear whether physiological changes that occur with overexpression of *TaGATA38* are related to increased susceptibility to WSS in Reeder and lines with the *3BLa* allele.

In the spring wheat dataset, *TraesCS3B02G597900* (*TaVPE3cB*) was significantly upregulated at Zadoks 59 across all lines. Increased expression was also observed in hollow stemmed lines with the *3BLa* allele (**Supplementary Figure 2G**). Recently, *TraesCS3B02G597900* was identified as a candidate gene for pith thickness at plant maturity by Liu et al. (2023) and was named *TaVPE3cB*. Similar to our results, these authors found that this gene showed significant expression differences between the early stem elongation stage (Zadoks 32) and mid-anthesis (Zadoks 65) with the highest expression in stems of a low pith thickness/hollow cultivar (Liu et al., 2023). *TaVPE3cB* is a member of the VPE3 gene family and codes for a vacuolar processing enzyme (VPE) that is highly expressed in stem tissues at the stem elongation stage (Liu et al., 2023). In monocots, VPEs are often classified into two subgroups, one group contains VPE genes which are expressed in seeds and the other contains genes which are specific to leaves and stems. While there are exceptions, VPEs found in leaves and stems (α-VPE and γ-VPE) are generally associated with lysis while seed type VPEs (β-VPEs) are associated with storing various types of proteins (Vorster et al., 2019). In Arabidopsis, γ-VPE was found to be expressed in the xylem and phloem of developing stems and is involved in programmed cell death (PCD) and thickening of secondary cell walls through activation of CEP1 proteases (Cheng et al., 2019). It is therefore possible that the increased expression of *TaVPE3cB* in hollow stemmed lines at the later growth stage may be responsible for PCD and the hollow stemmed phenotype observed.

### 4.5 Other durum wheat genes

*TdDof* (*TRITD3Bv1G280530*), coding for a Dof zinc finger protein, was previously found to be responsible for the majority of variation in stem solidness in both durum and spring wheat mapping populations and is a strong candidate gene for *SSt1*. Increased copy number variation (CNV) of this gene observed was observed in solid stemmed lines, with the exception of a single solid stemmed Australian cultivar ‘Janz’ which had just one copy (Nilsen et al., 2020, 2017). Their results suggest regulation of genes involved in programmed cell death by *TdDof* during early stem elongation in hollow stemmed cultivars leading to loss of pith parenchyma tissue (Nilsen et al., 2020). While our study focused on the 3A QTL in durum wheat associated with early stem solidness and not the 3B QTL where *SSt1* is located, an increase in transcripts of *SSt1* was observed in the parental lines although this difference was not statistically significant. Between Zadoks stage 49 and 77, the early stem solidness observed in Pierce during early stem elongation (Zadoks 34-37) disappears, leaving it with stem solidness similar to susceptible cultivars of both durum and spring wheat (Varella et al., 2019b). In our study, we observed that the transcript abundance of *SSt1* decreased with plant maturity in both Pierce and PI 41353. Expression was slightly higher in solid stemmed Pierce samples although neither allele nor growth stage had a significant effect on the gene expression of *SSt1* (**Supplementary Figure 9I**).

Other research identified *TraesCS3B02G608800*, an ortholog of *TRITD3Bv1G280530* in spring wheat, as *TaDof3.2-3B*. They found that expression of this gene was generally low in several cultivars of spring wheat and in most plant tissues did not change much through plant development or in response to biotic and abiotic stresses including infection with *F. graminearum* or drought, heat and cold stress (Fang et al., 2020). It was found that *TaDof3.2-3B* was highly expressed at early stem elongation, especially in the solid stemmed cultivar ‘Westonia’, an expression pattern that was also observed in durum wheat (Liu et al., 2023; Nilsen et al., 2020). In our results, expression of *TaDof3.2-3B* also decreased from Zadoks 49 to 59 in hollow stemmed Reeder and NILs with the *3BLa* allele (Data not shown). However, Conan displayed a slight increase in expression from Zadoks 49 to 59 and NILs with the *3BLc* allele experienced a slight decrease in transcript abundance at the later growth stage. Conan also had lower expression of *TaDof3.2-3B* than Reeder, indicating that this gene may not be solely responsible for the early stem solidness phenotype seen in Conan. While stem solidness continues to decrease in Conan from Zadoks 49 to head emergence (Zadoks 59), the loss of stem solidness from early stem elongation (Zadoks 35) to head emergence is much more drastic, a trend also observed in the durum wheat cultivar Pierce which also exhibits early stem solidness (Varella, 2016; Varella et al., 2019a). This means that it is possible that Conan and Pierce may have higher expression of *SSt1* at the start of stem elongation that we were unable to capture as it occurred prior to our collection of stem samples at Zadoks 49.

In the durum wheat dataset *TRITD3Av1G185620*, an isoleucine-tRNA ligase and *TRITD3Av1G185910*, a MADS-box transcription factor, were significantly upregulated in the hollow stemmed cultivar PI 41353 as well as NILs with the *3ALa* allele (**Figures 6A, B**). These transcripts were also found in higher abundance in hollow stemmed lines and *pithless1* mutants in a previous whole genome sequencing analysis aimed at identifying candidate genes for *SSt1*, although the differences were not found to be significant (Nilsen et al., 2020). This indicates that in durum wheat, isoleucine-tRNA ligase and MADS-box transcription factors may be associated with the hollow stemmed phenotype, at least in early development. Although MADS-box transcription factors have not been well characterized in durum wheat, genome wide identification and characterization of this gene family has been performed in bread wheat, most recently with the publicly available reference genome IWGSC Ref. Seq v1.1 (Ma et al., 2017; Raza et al., 2022). The durum wheat gene MADS-box transcription factor shares 100% sequence homology with hexaploid wheat gene *TraesCS3A02G284400*, a MIKC-type MADS-box gene that is typically highly expressed in the apical meristem and is known to be involved in regulation of the flowering stage (Yang et al., 2021). A strong positive correlation between this gene and abundance of the phenolic compound quercetin-3-O-rutinoside (rutin) has also been observed in hexaploid wheat, pointing at an association between this gene and high antioxidant activity (Ma et al., 2022). While rutin was not found in the durum wheat dataset, analysis of the complete spring wheat dataset revealed that growth stage had a significant effect on the related rutinoside cyanidin-3-rutinoside. Phenolic compounds and other flavonoids are reported to be involved with feeding and oviposition behaviors in many plant-insect systems (Simmonds, 2001), and rutin in particular is associated with increased feeding and oviposition in many insect species including *Heliothis zea* and *Helicoverpa armigera*, although behavioral response can vary depending on the growth stage of the insect and flavonoid concentration (Blaney and Simmonds, 1983; Ohsugi et al., 2014). Rutin has also been shown to inhibit the growth and survivorship of European gypsy moth *Lymantria dispar* L. and European corn borer *Ostrina nubilalis* Hübner (Beninger and Abou-Zaid, 1997; Abou-Zaid et al., 1993). Further characterization of the effects of these genes in hollow stemmed durum and spring wheat cultivars may determine how they are associated with susceptible phenotypes.

## 5 Conclusion

While the solid stem phenotype is widely used to protect against crop loss due to wheat stem sawfly, expression of solid stems can be variable depending on environmental conditions. Additionally, braconid parasitoid populations are also negatively affected by the continued use of solid stemmed cultivars. These drawbacks mean that despite the success of solid stemmed cultivars as a control measure against WSS, extensive yield loss and stem cutting can still occur, and it is necessary to explore additional sources of resistance such as the early stem solidness phenotype. Early stem solidness is a phenotype that offers protection to the plant through the time of flight and oviposition period of WSS, while also allowing for better support of populations of braconid parasitoids since pith is lost as the plant develops. Assessing transcriptomic and metabolomic responses in cultivars with early stem solidness such as the spring wheat cultivar Conan and the durum wheat cultivar Pierce will help elucidate the mechanisms related to the early stem solidness phenotype and lead toward development of cultivars that can be used to strengthen the tritrophic interaction between wheat, WSS and their parasitoids and prevent yield loss.

Here we identified effects of growth stage and allele on expression of metabolites and transcripts associated with the phenylpropanoid pathway. In the complete spring wheat dataset a caffeic acid methylesterase and a pectin methylesterase were both upregulated in Reeder and lines with the *3BLa* allele, suggesting that these genes are likely affecting total lignin content, lignin subunit proportion, cell wall accessibility of pectin or production of volatile semiochemicals such as (Z)-3-hexenyl acetate that affect WSS behavior. Several other studies involving stem solidness or the WSS tritrophic system have implicated metabolites, proteins and genes associated with the phenylpropanoid pathway, however expression is affected by genotype and is highly variable even in the absence of WSS infestation. While the role of the phenylpropanoid pathway in WSS resistance has yet to be fully characterized, these potential structural differences in stem tissue on the molecular level are likely responsible for the resistance of some cultivars to WSS infestation and should be explored further.

Differences in carbohydrate abundance were observed in both the spring and durum wheat datasets, but the patterns of abundance varied depending on carbohydrate type, growth stage and line. WSS infestation has been shown to have an effect on carbohydrate abundance, where responses differ depending on cultivar and specific carbohydrate type (Lavergne et al., 2020; Biyiklioglu et al., 2018). In this study, water soluble carbohydrates (WSC) were found in higher abundance in solid stemmed cultivars, matching previous associations of WSC content with stem solidness in uninfested plants (Nilsen et al., 2020). WSS larvae feed on parenchyma tissue which is where the majority of carbohydrates are located inside the stem, and despite the variability in carbohydrate concentration between cultivars, the ratio of proteins to carbohydrates in consumed tissue can affect larval development in a variety of ways (Holmes, 1949; Ford et al., 1979). Effects of carbohydrates in the diets of WSS larvae can also carry over to other trophic levels and influence the fitness of future parasitoid generations as observed in other species (Roeder et al., 2014). Unfortunately, protein:carbohydrate ratios in insect diets can have either positive or negative effects depending on species and specific concentrations of proteins and carbohydrates so additional investigation is needed to determine whether there are differences in the protein:carbohydrate ratio of stem tissue in resistant lines and the effects these ratios might have on WSS growth and development.

Growth stage and line both had significant effects on lipids identified in the spring and durum wheat datasets. In spring wheat, an intermediate of the sphingolipid biosynthesis pathway, 1-phosphatidyl-1D-myo-inositol 3-phosphate, increased at the later growth stage while an intermediate of glycerolipid biosynthesis, glycerol-3-phosphate decreased. The durum wheat dataset also showed that several phospholipids decreased with plant maturity in the resistant line. Induced changes to the lipidome and the subsequent effects on insect pests are highly variable in other plant-insect interactions but can affect larval development (Lehmann et al., 2020; Sinclair and Marshall, 2018; Li et al., 2014; Liao et al., 2021). The effect of the lipidome on WSS larvae has not been characterized, but due to differences observed in this study between resistant and susceptible lines, it is possible that lipid profiles are associated with resistance or susceptibility to WSS.

From our transcriptome analysis, genes associated with stem solidness, programmed cell death and cell wall development were also identified, including *TaVPE3cB*, *TdDof* and *TaDof*. Increased expression of *TaVPE3cB*, a gene associated with increased programmed cell death and cell wall thickening was observed in Reeder and lines with the *3BLa* allele indicating that this gene is likely responsible for the hollow stemmed phenotype in Reeder. *TdDof*, previously identified as a candidate gene for *SSt1* in durum wheat, was not differentially expressed between hollow stemmed lines and lines with the early stem solidness allele in our study. *TaDof*, ortholog of *TdDof* in spring wheat, decreased at the later growth stage in Reeder and lines with the *3BLa* allele, while Conan experienced a slight decrease and overall lower expression than the hollow stemmed lines. Most solid stemmed cultivars have increased copy number variation of *TdDof*, however the expression observed in Pierce and Conan seems to suggest that *TdDof/TaDof* is not the only gene responsible for the early stem solidness trait.

Although there is high variability in the metabolome, proteome and transcriptome of solid stemmed cultivars, hollow stemmed cultivars and cultivars that exhibit early stem solidness, there is a consensus that the phenylpropanoid pathway, carbohydrate biosynthesis and programmed cell death are likely involved in resistance and plant response to WSS infestation. Differences in metabolites and genes involved in lignin biosynthesis and subunit composition as well as variability in carbohydrates and lipids demonstrate the need for further characterization of molecular differences in the stem architecture of resistant cultivars as well as the effect of tissue composition on the behavior and development of WSS. Additionally, further exploration of the 3B QTL and previously identified candidate genes for *SSt1* will help identify the mechanism responsible for regulating early stem solidness.

## Data Availability

The datasets presented in this study can be found in the National Center for Biotechnology Information Sequence Read Archive (SRA) database (https://www.ncbi.nlm.nih.gov/sra; accession no. PRJNA1111838).

## Supplementary material

Supplementary Table 1 Scores from principal component analysis (PCA) on spring wheat dataset.

Supplementary Table 2 Eigenvalues from principal component analysis (PCA) on spring wheat dataset.

Supplementary Table 3 Loadings from principal component analysis (PCA) on spring wheat dataset.

Supplementary Table 4 Complete list of annotated gene transcripts in spring wheat with significance of allelic differences.

Supplementary Table 5 Fold changes of spring wheat transcripts.

Supplementary Table 6 Complete list of annotated gene transcripts in spring wheat with significance of stage differences.

Supplementary Table 7 KEGG enrichment *3BLc49* vs *3BLa49*.

Supplementary Table 8 KEGG enrichment *3BLc59* vs *3BLa59*.

Supplementary Table 9 Complete list of metabolite features in spring wheat.

Supplementary Table 10 Annotations of identified metabolites in the complete spring wheat dataset which showed significance in two-way ANOVA.

Supplementary Table 11 Fold changes of spring wheat metabolites.

Supplementary Table 12 Results from ANOVA post-hoc test, Fisher’s LSD on spring wheat dataset.

Supplementary Table 13 Annotations of identified metabolites in the NIL only dataset which showed significance in two-way ANOVA.

Supplementary Table 14 Annotations of identified metabolites which showed significance between *3BLb* and *3BLc* samples in the two-way ANOVA.

Supplementary Table 15 Annotations of identified metabolites which showed significance between *3BLb* and *3BLa* samples in the two-way ANOVA.

Supplementary Table 16 Scores from principal component analysis (PCA) on durum wheat dataset.

Supplementary Table 17 Eigenvalues from principal component analysis (PCA) on durum wheat dataset.

Supplementary Table 18 Loadings from principal component analysis (PCA) on durum wheat dataset.

Supplementary Table 19 Complete list of annotated gene transcripts in durum wheat with significance of allelic differences.

Supplementary Table 20 Fold changes of durum wheat transcripts.

Supplementary Table 21 Complete list of annotated gene transcripts in durum wheat with significance of stage differences.

Supplementary Table 22 KEGG enrichment *3ALb49* vs *3ALa49*.

Supplementary Table 23 KEGG enrichment *3ALb59* vs *3ALa59*.

Supplementary Table 24 Complete list of metabolite features in durum wheat.

Supplementary Table 25 Annotations of identified metabolites in the complete durum wheat dataset which showed significance in two-way ANOVA.

Supplementary Table 26 Fold changes of durum wheat metabolites.

Supplementary Table 27 Results from ANOVA *post-hoc* test, Fisher’s LSD on durum wheat dataset.
