# Supplementary material for: Untargeted metabolomic and transcriptomic analysis in spring and durum wheat reveals potential mechanisms associated with the early stem solidness phenotype and resistance to wheat stem sawfly
Source: Front Plant Sci. 2025 Feb 19;16:1497732. doi: 10.3389/fpls.2025.1497732 (PMC11880032; doi:10.3389/fpls.2025.1497732)
Supplement: Supplementary file 3 [file DataSheet1.docx]

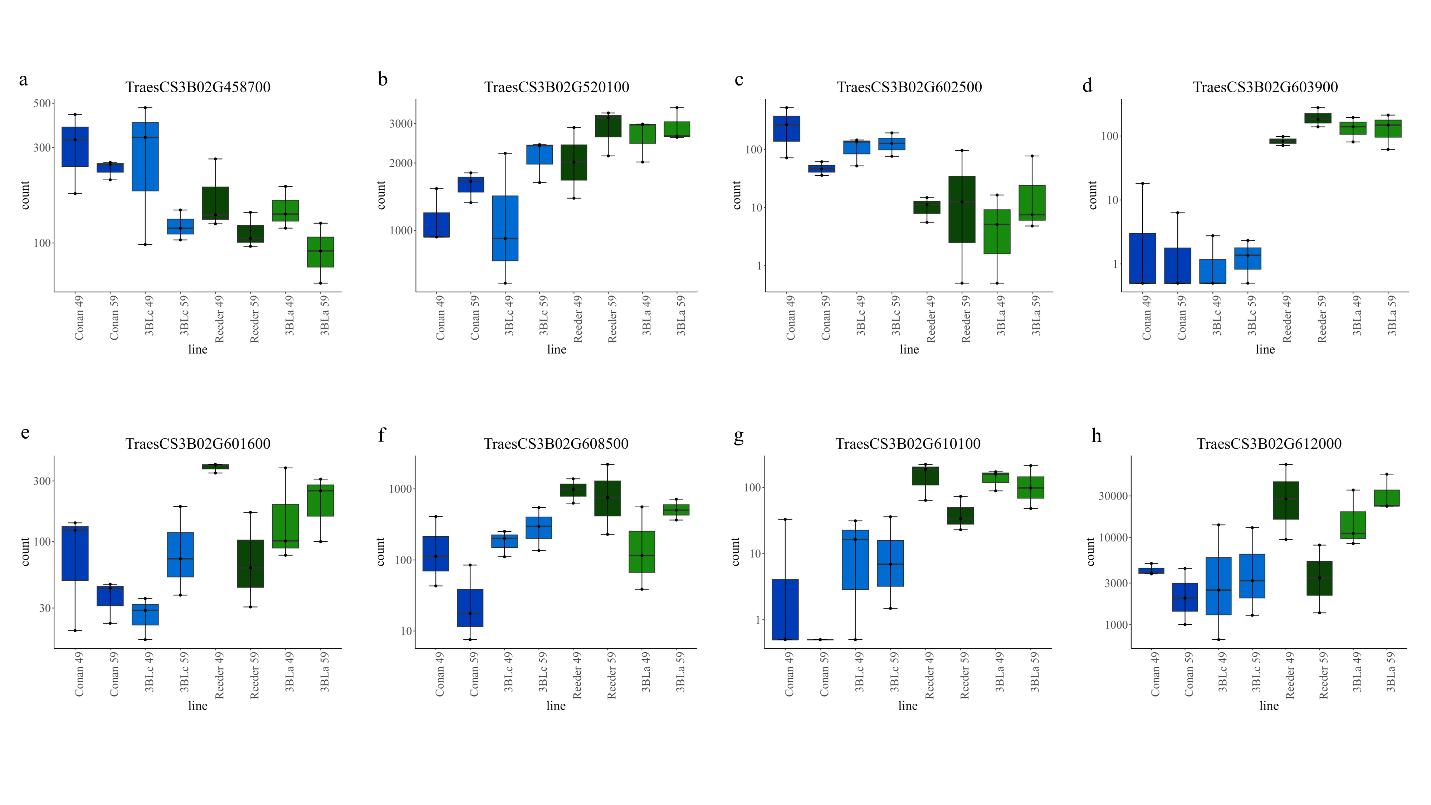


Supplementary Figure 1. Boxplots showing differential expression of spring wheat transcripts with significant effects of allele in NILs of spring wheat. The box indicates the interquartile range (IQR) with the horizontal bar indicating the median. Lines extending from the box show the standard error for each sample group. Conan, dark blue; *3BLc*, royal blue; Reeder, dark green; *3BLa*, green.


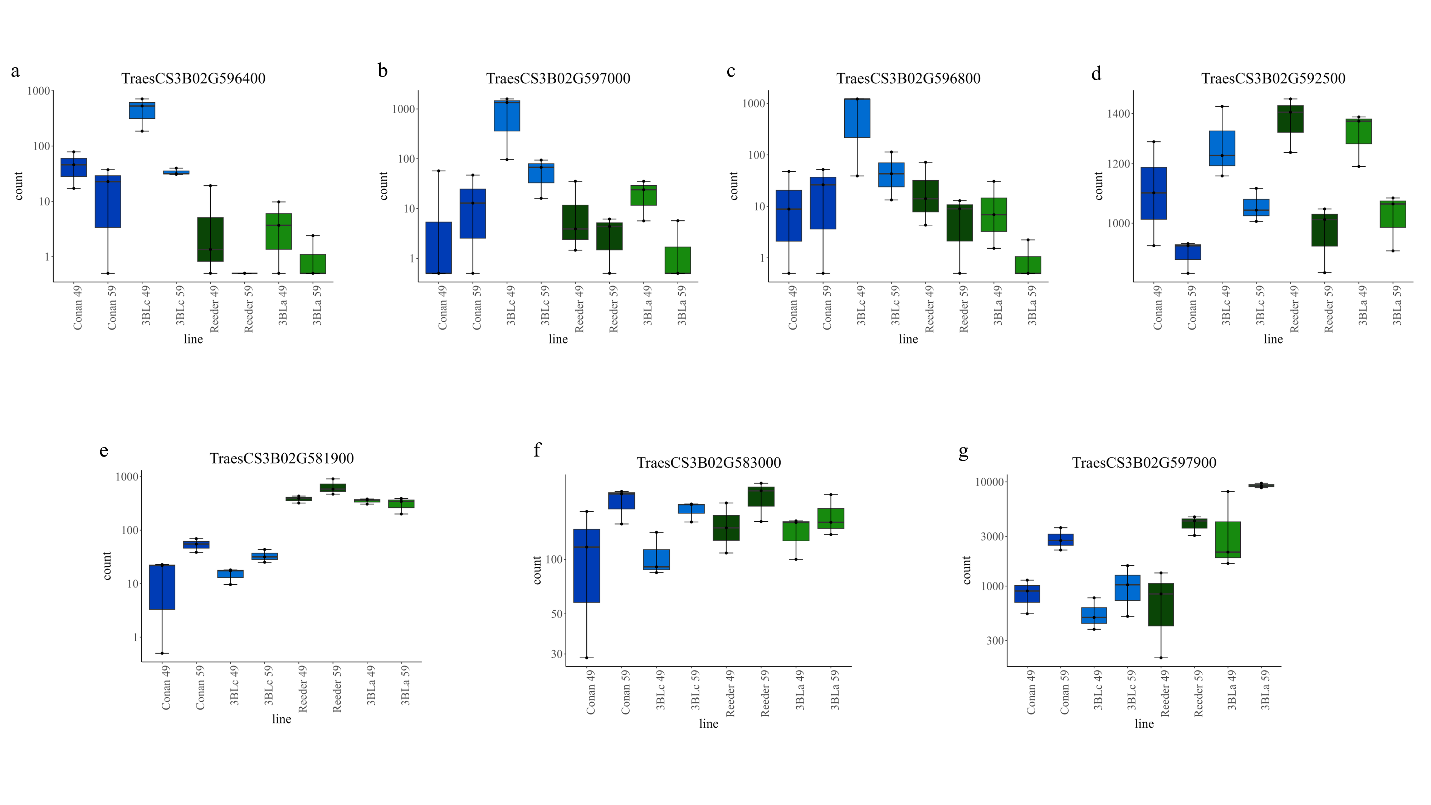


Supplementary Figure 2. Boxplots showing differential expression of spring wheat transcripts with significant effects of growth stage in NILs of spring wheat. The box indicates the interquartile range (IQR) with the horizontal bar indicating the median. Lines extending from the box show the standard error for each sample group. Conan, dark blue; *3BLc*, royal blue; Reeder, dark green; *3BLa*, green.


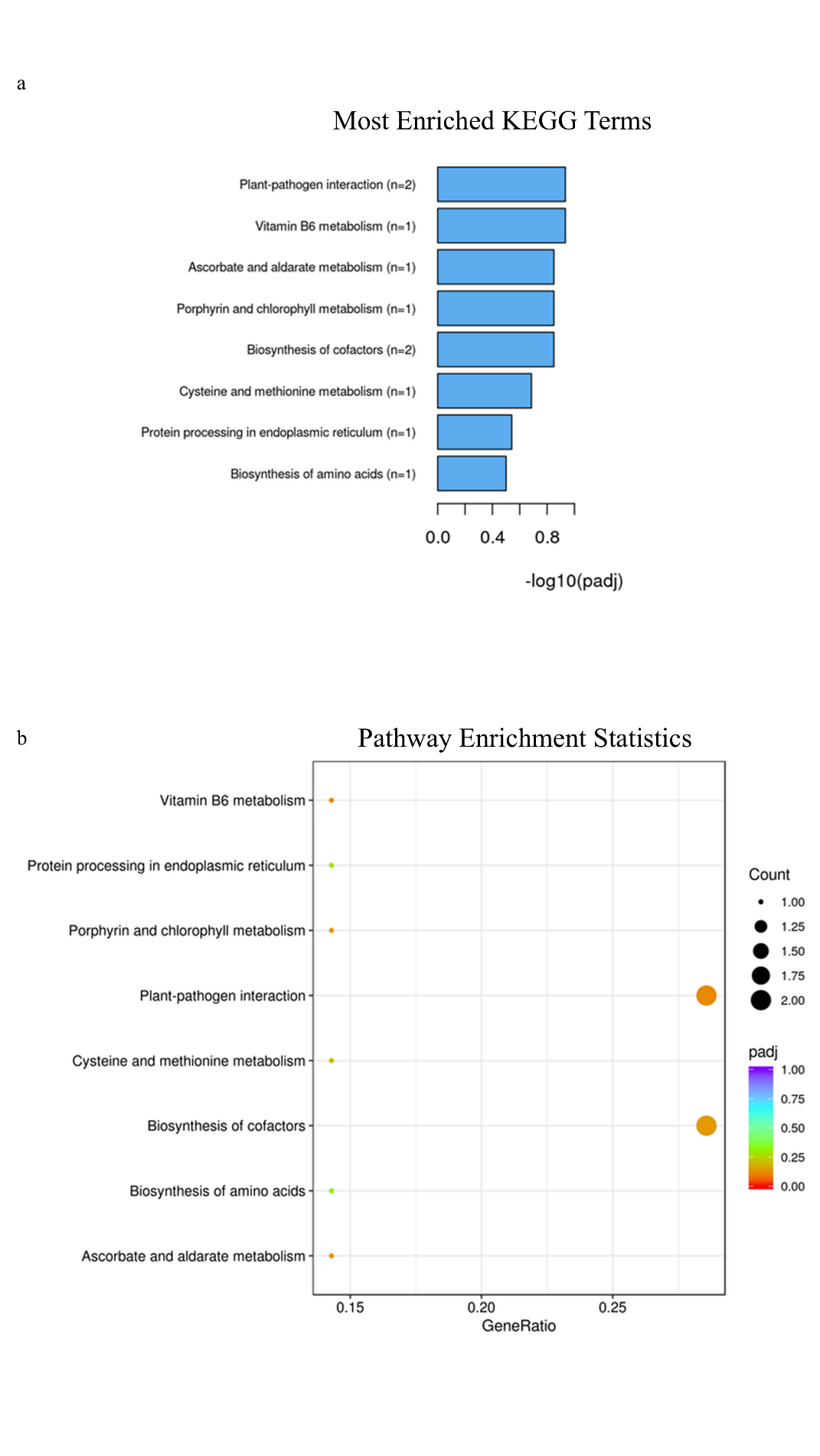


Supplementary Figure 3. KEGG enrichment histogram of the top 20 significantly enriched terms in KEGG enrichment analysis of spring wheat samples with the 3BLc and 3BLa alleles at Zadoks 59.


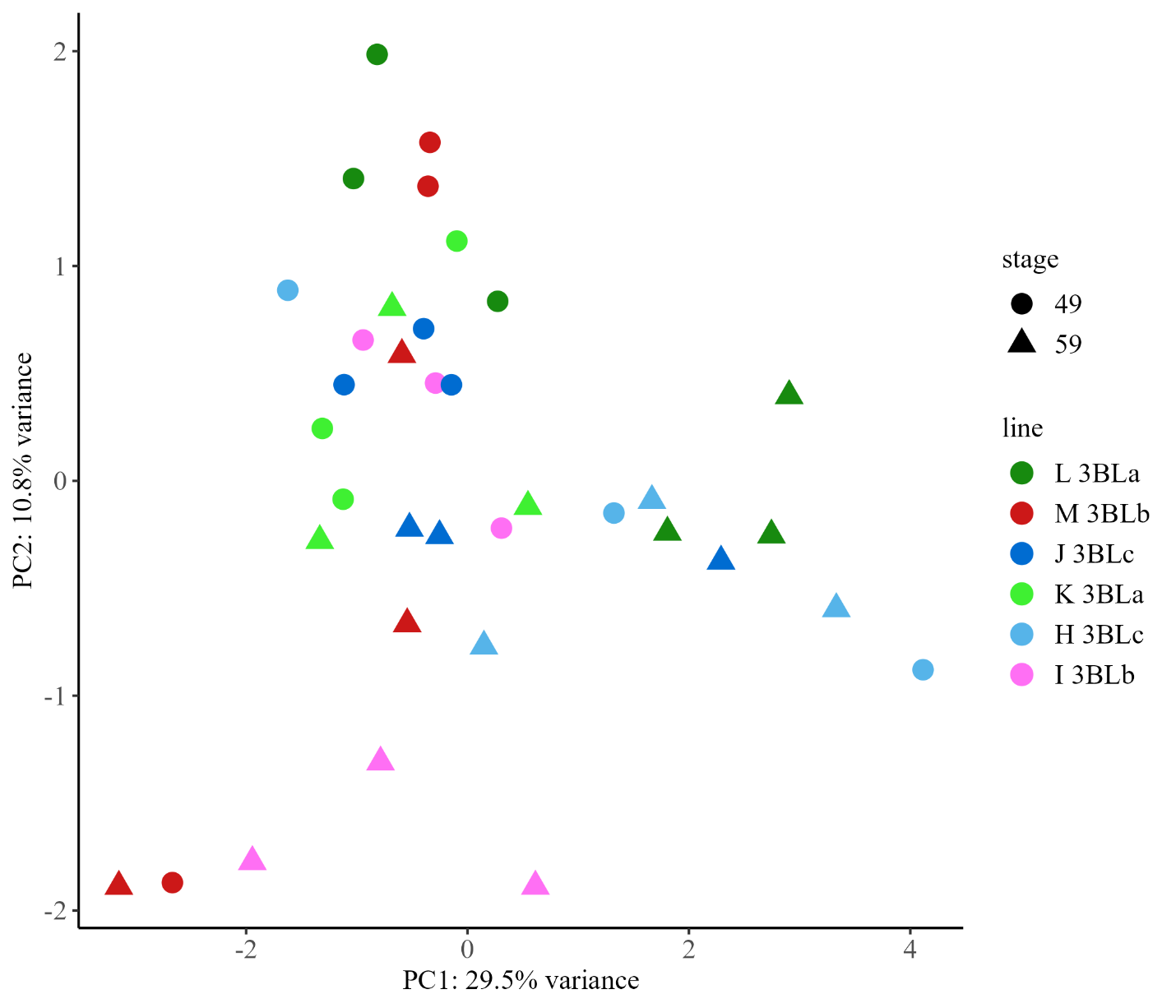


Supplementary Figure 4. Principal component analysis (PCA) score plots for early (Zadoks 49) and late (Zadoks 59) stage samples of spring wheat metabolites from near isogenic lines with *3BLa*, *3BLb* and *3BLc* alleles. PCA plots were created using LC-MS data from early and late stage plants with each point representing a sample from a main stem. L *3BLa*, green; M *3BLb*, red; J *3BLc*, royal blue; K *3BLa*, light green; H *3BLc*, light blue; I *3BLb*, pink; early, circle; late, triangle.


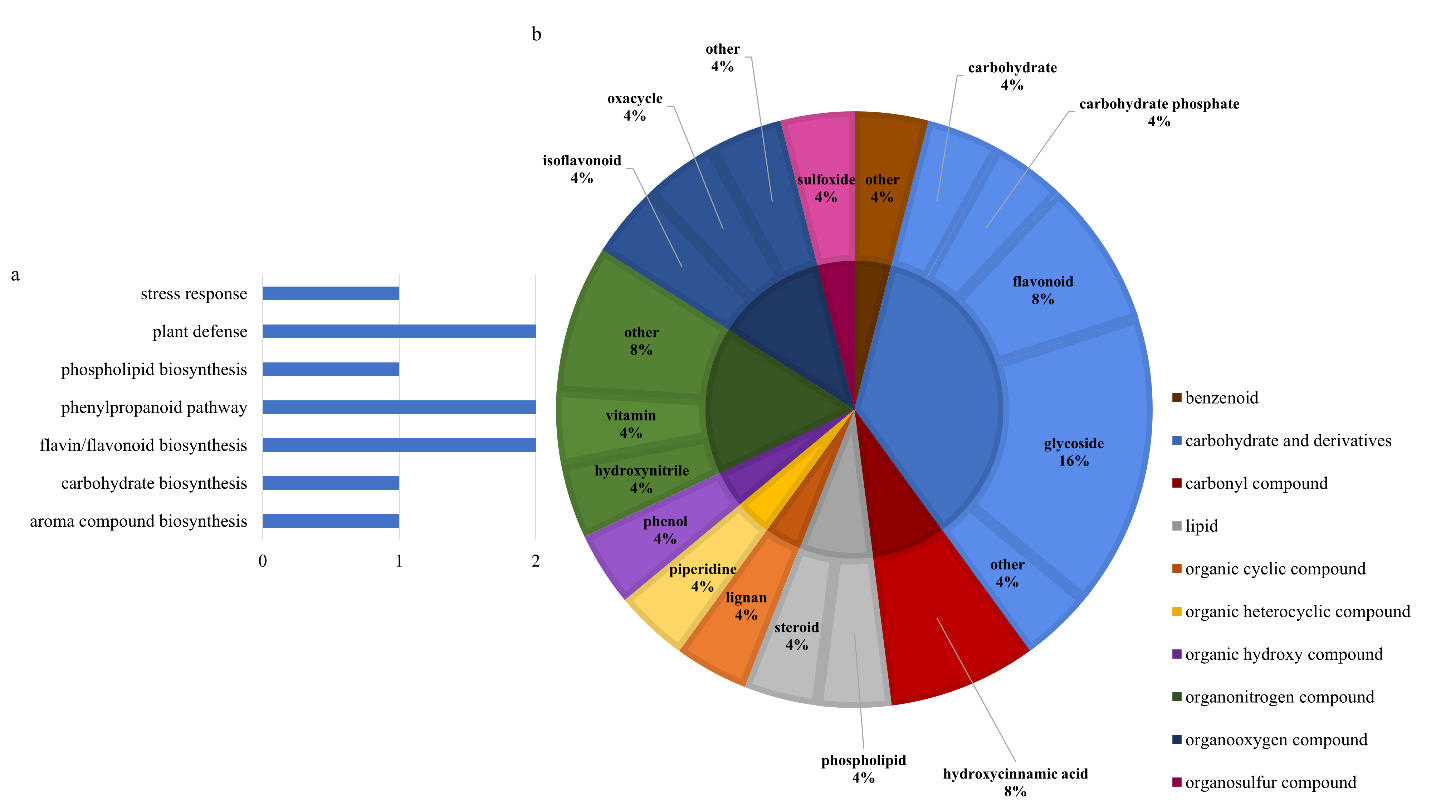


Supplementary Figure 5. A) Biochemical pathway associations of significant compounds in the spring wheat NIL dataset which consisted of all samples from near isogenic lines. Significant compounds (p-values<0.05) were identified using two-way ANOVA. B) Classifications of significant putatively identified metabolites in the spring wheat dataset. The innermost circle indicates the compound class with the outer ring representing compound subclass. Carbohydrate and derivatives, royal blue; carbonyl compound, red; lipid, grey; organic heterocyclic compound, yellow; organic hydroxy compound, purple; organonitrogen compound, dark green; organooxygen compound, dark blue; other, brown.


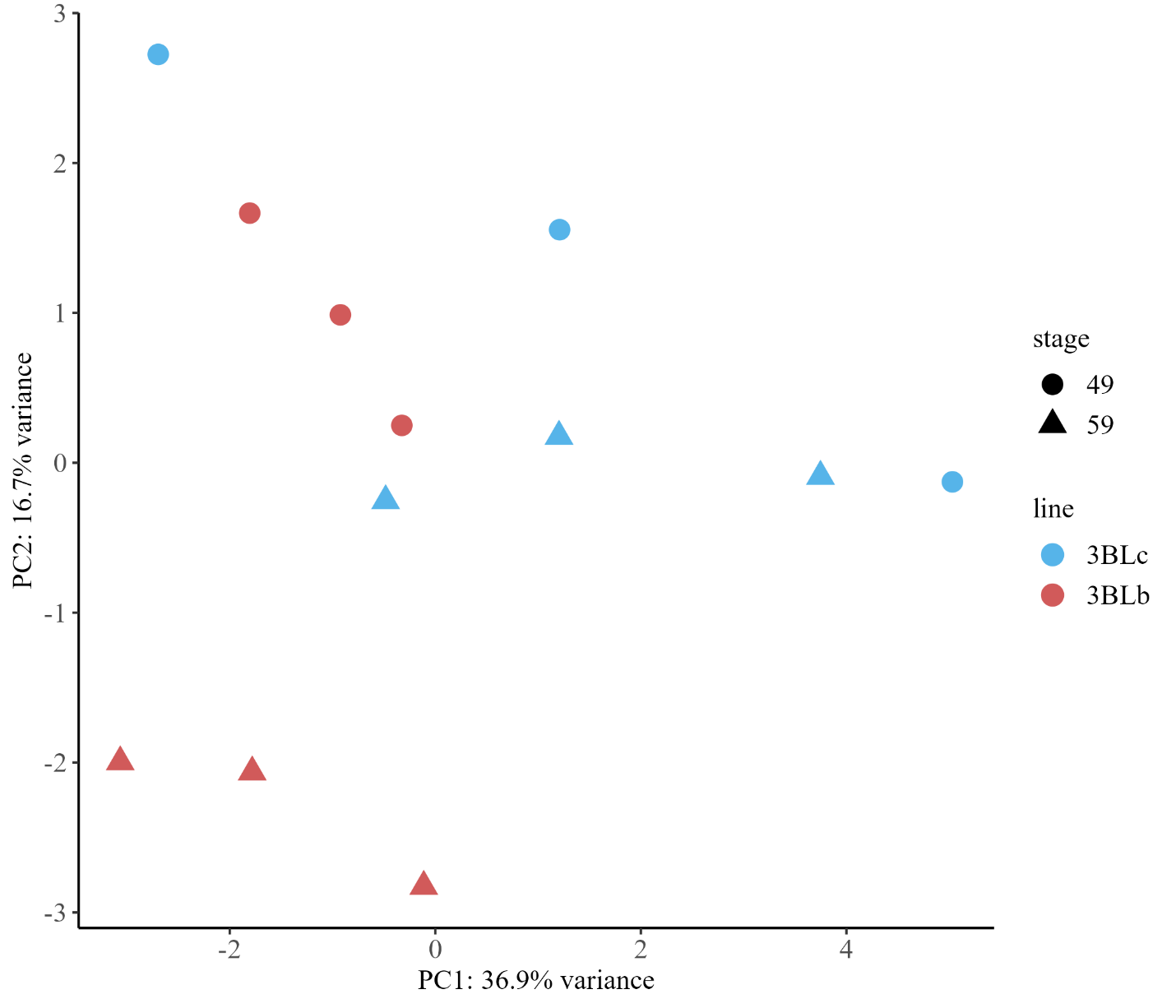


Supplementary Figure 6. Principal Component Analysis (PCA) score plots for early (Zadoks 49) and late (Zadoks 59) stage samples of spring wheat metabolites from near isogenic lines with *3BLb* and *3BLc* alleles. PCA plots were created using LC-MS data from early and late stage plants with each point representing a sample from a main stem. *3BLc*, light blue; *3BLb*, red; early, circle; late, triangle.


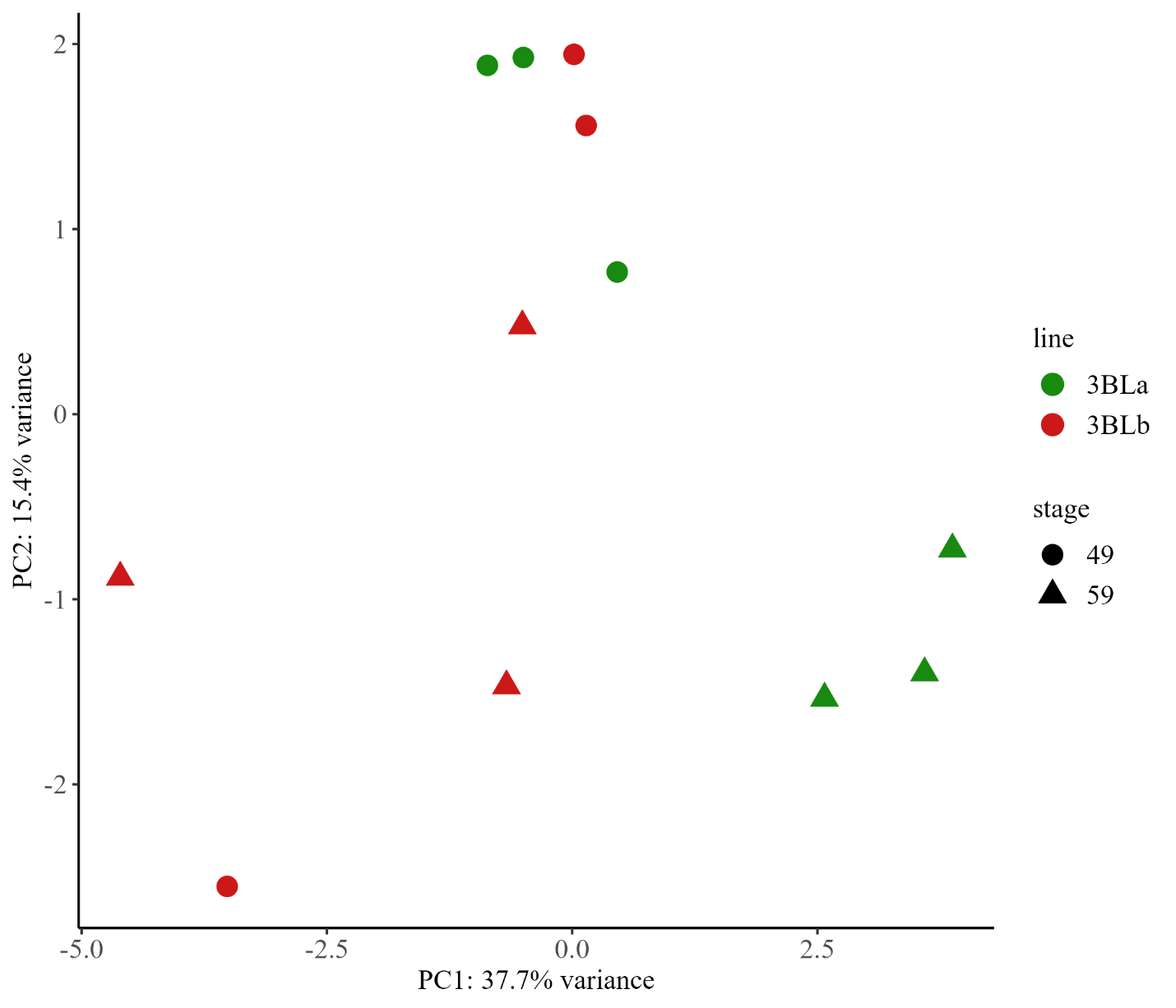


Supplementary Figure 7. Principal Component Analysis (PCA) score plots for early (Zadoks 49) and late (Zadoks 59) stage samples of spring wheat metabolites from near isogenic lines with *3BLa* and *3BLb* alleles. PCA plots were created using LC-MS data from early and late stage plants with each point representing a sample from a main stem. *3BLb*, red; *3BLa*, green; early, circle; late, triangle.


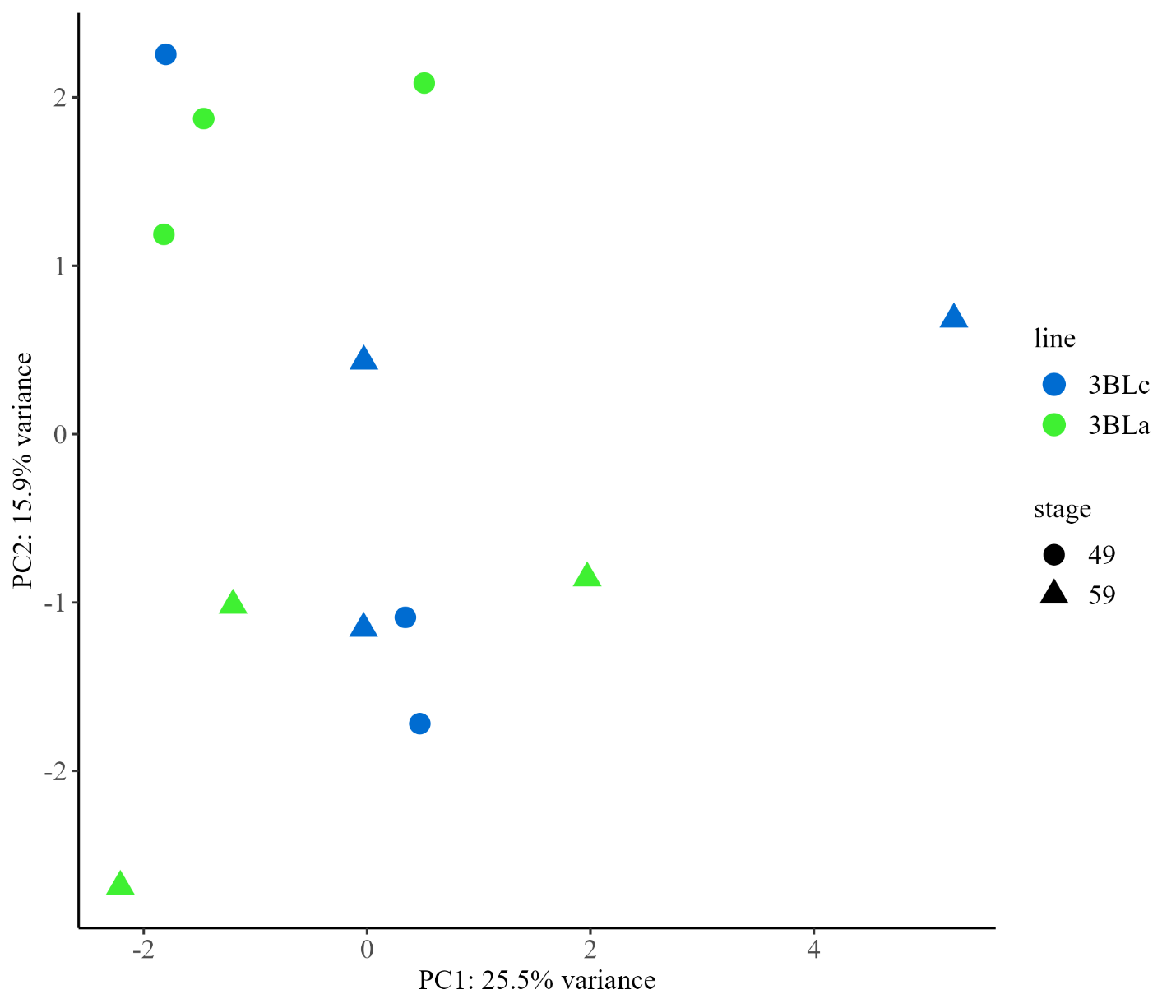


Supplementary Figure 8. Principal Component Analysis (PCA) score plots for early (Zadoks 49) and late (Zadoks 59) stage samples of spring wheat metabolites from near isogenic lines with *3BLa* and *3BLc* alleles. PCA plots were created using LC-MS data from early and late stage plants with each point representing a sample from a main stem. *3BLc*, royal blue; *3BLa*, light green; early, circle; late, triangle.


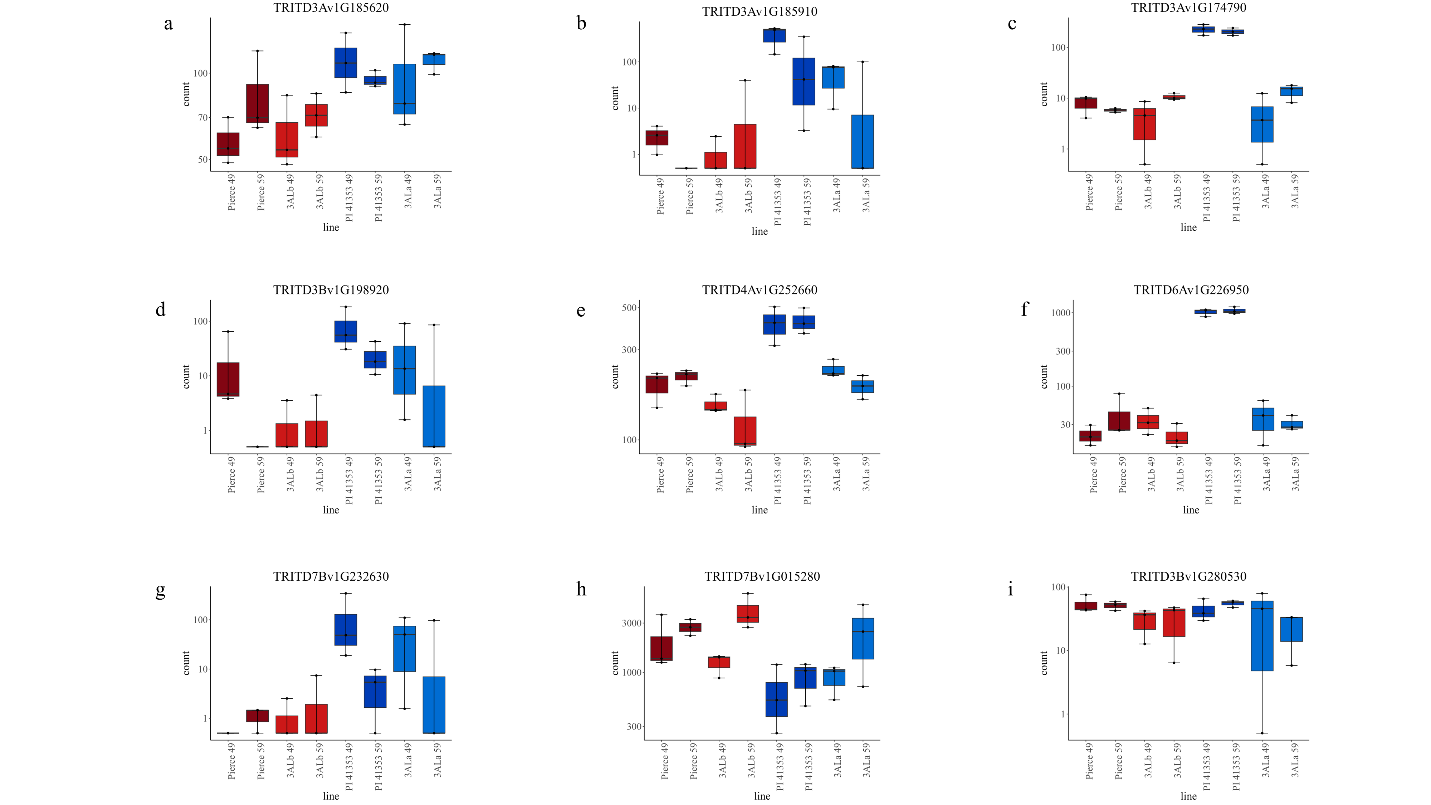


Supplementary Figure 9. Boxplots showing differential expression of durum wheat transcripts with significant effects of allele. The box indicates the interquartile range (IQR) with the horizontal bar indicating the median. Lines extending from the box show the standard error for each sample group. Pierce, maroon; *3ALb*, red; PI 41353, dark blue; *3ALa*, royal blue


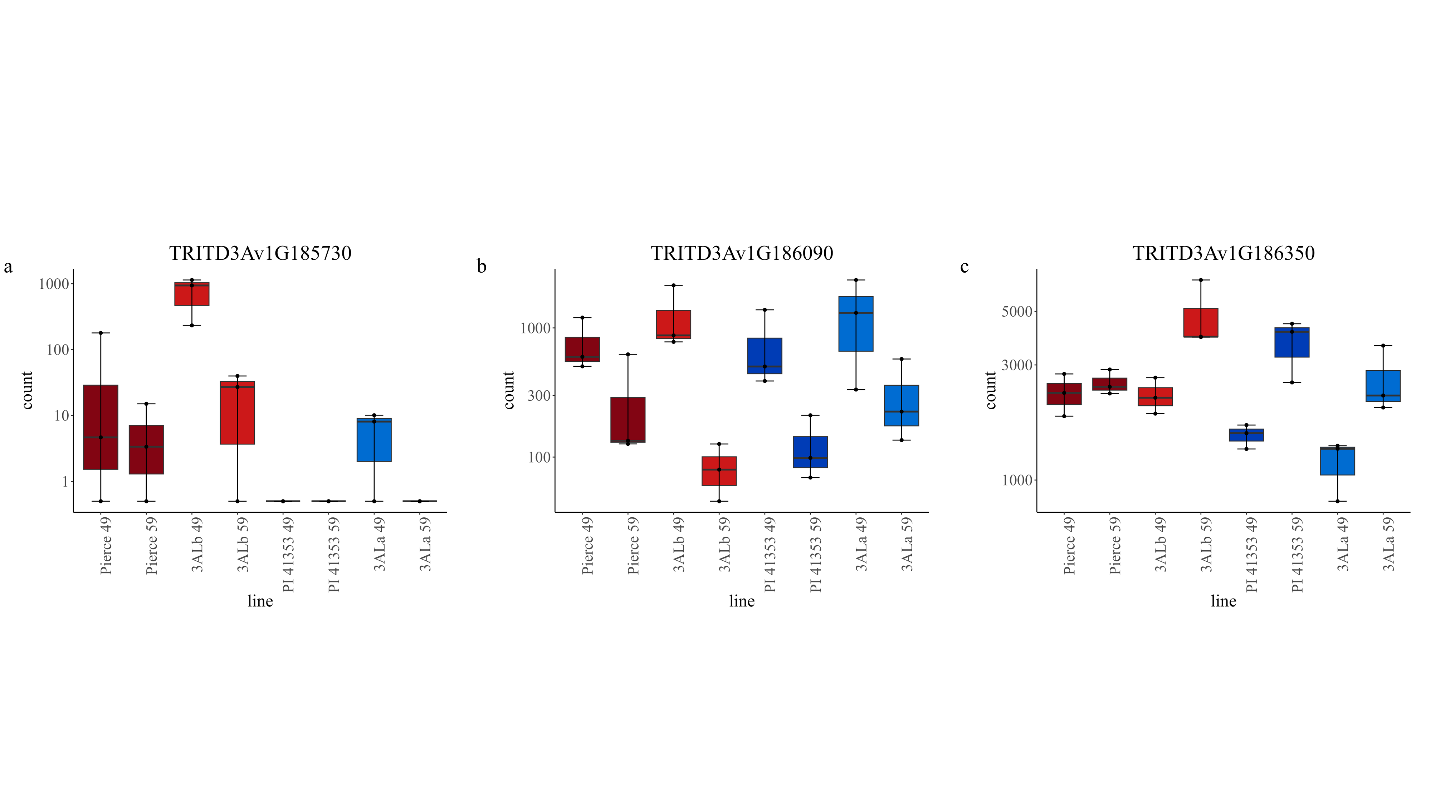


Supplementary Figure 10. Boxplots showing differential expression of durum wheat transcripts with significant effects of growth stage. The box indicates the interquartile range (IQR) with the horizontal bar indicating the median. Lines extending from the box show the standard error for each sample group. Pierce, maroon; *3ALb*, red; PI 41353, dark blue; *3ALa*, royal blue.


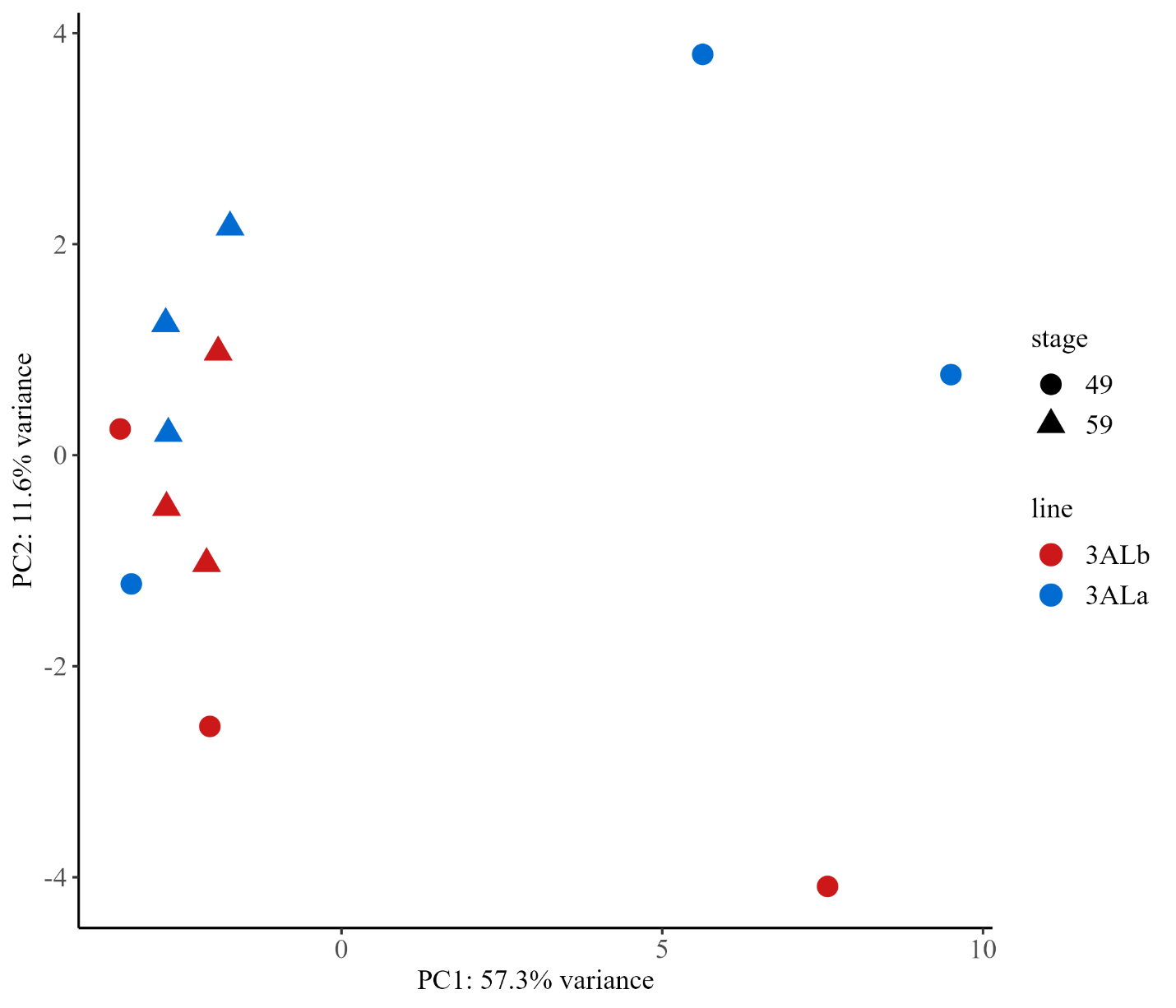


Supplementary Figure 11. Principal component analysis (PCA) score plots for early (Zadoks 49) and late (Zadoks 59) stage samples of durum wheat metabolites from near isogenic lines with *3ALb* and *3ALa* alleles. PCA plots were created using LC-MS data from early and late stage plants with each point representing a sample from a main stem. *3ALb*, red; *3ALa*, royal blue; early, circle; late, triangle.
